# Brain Hypoxia Is Associated With Neuroglial Injury in Humans Post–Cardiac Arrest

**DOI:** 10.1161/CIRCRESAHA.121.319157

**Published:** 2021-07-21

**Authors:** Ryan L. Hoiland, Philip N. Ainslie, Cheryl L. Wellington, Jennifer Cooper, Sophie Stukas, Sonny Thiara, Denise Foster, Nicholas A. Fergusson, Edward M. Conway, David K. Menon, Peter Gooderham, Veronica Hirsch-Reinshagen, Donald E. Griesdale, Mypinder S. Sekhon

**Affiliations:** 1 Department of Anesthesiology, Pharmacology and Therapeutics, Vancouver General Hospital (R.L.H., D.E.G.), University of British Columbia, Vancouver, Canada.; 2 Department of Cellular and Physiological Sciences, Faculty of Medicine (R.L.H.), University of British Columbia, Vancouver, Canada.; 3 Centre for Heart, Lung, and Vascular Health, School of Health and Exercise Sciences, Faculty of Health and Social Development (R.L.H., P.N.A.), University of British Columbia, Vancouver, Canada.; 4 Djavad Mowafaghian Centre for Brain Health, Department of Pathology and Laboratory Medicine, Faculty of Medicine (C.L.W., J.C., S.S.), University of British Columbia, Vancouver, Canada.; 5 Division of Critical Care Medicine, Department of Medicine, Vancouver General Hospital (S.T., D.F., N.A.F., D.E.G., M.S.S.), University of British Columbia, Vancouver, Canada.; 6 Centre for Blood Research, Life Sciences Institute, University of British Columbia (E.M.C.), University of British Columbia, Vancouver, Canada.; 7 Division of Neurosurgery, Department of Surgery, Vancouver General Hospital (P.G.), University of British Columbia, Vancouver, Canada.; 8 Centre for Clinical Epidemiology and Evaluation, Vancouver Coastal Health Research Institute (D.E.G.), University of British Columbia, Vancouver, Canada.; 9 School of Biomedical Engineering (C.L.W.), University of British Columbia, Vancouver, Canada.; 10 Division of Hematology, Department of Medicine (E.M.C.), University of British Columbia, Vancouver, Canada.; 11 International Collaboration on Repair Discoveries, Vancouver, BC, Canada (R.L.H., C.L.W., J.C., S.S., V.H.-R.).; 12 Department of Clinical Neurosciences, Addenbrookes Hospital, University of Cambridge (D.K.M.).

**Keywords:** biomarker, brain injury, healthy volunteers, inflammation, partial pressure

## Abstract

Secondary brain hypoxia portends significant mortality in ischemic brain diseases; yet, our understanding of hypoxic ischemic brain injury (HIBI) pathophysiology in humans remains rudimentary.

**Objective:**

To quantify the impact of secondary brain hypoxia on injury to the neurovascular unit in patients with HIBI.

**Methods and Results:**

We conducted a prospective interventional study of invasive neuromonitoring in 18 post–cardiac arrest patients with HIBI. The partial pressures of brain tissue O_2_ (PbtO_2_) and intracranial pressure were directly measured via intraparenchymal microcatheters. To isolate the cerebrovascular bed, we conducted paired sampling of arterial and jugular venous bulb blood and calculated the transcerebral release of biomarkers of neurovascular injury and inflammation in the patients with HIBI and 14 healthy volunteers for control comparisons. Ten patients with HIBI exhibited secondary brain hypoxia (PbtO_2_<20 mmHg), while 8 exhibited brain normoxia (PbtO_2_≥20 mmHg). In the patients with secondary brain hypoxia, we observed active cerebral release of glial fibrillary acidic protein (−161 [−3695 to −75] pg/mL; *P*=0.0078), neurofilament light chain (−231 [−370 to −11] pg/mL; *P*=0.010), total tau (−32 [−310 to −3] pg/mL; *P*=0.0039), neuron-specific enolase (−14 890 [−148 813 to −3311] pg/mL; *P*=0.0039), and ubiquitin carboxy-terminal hydrolase L1 (−14.7 [−37.7 to −4.1] pg/mL; *P*=0.0059) indicating de novo neuroglial injury. This injury was unrelated to the systemic global ischemic burden or cerebral endothelial injury but rather was associated with cerebral release of IL-6 (interleukin-6; −10.3 [−43.0 to −4.2] pg/mL; *P*=0.0039). No cerebral release of the aforementioned biomarkers was observed in patients with HIBI with brain normoxia or the healthy volunteers. Hyperosmolar therapy in the patients with secondary brain hypoxia reduced the partial pressure of jugular venous O_2_-to-PbtO_2_ gradient (39.6 [34.1–51.1] versus 32.0 [24.5–39.2] mm Hg; *P*=0.0078) and increased PbtO_2_ (17.0 [9.1–19.7] versus 20.2 [11.9–22.7] mm Hg; *P*=0.039) suggesting improved cerebrovascular-to-parenchymal O_2_ transport.

**Conclusions:**

Secondary brain hypoxia is associated with de novo neuroglial injury and cerebral release of IL-6. Mitigating cerebrovascular-to-parenchymal limitations to O_2_ transport is a promising therapeutic strategy for patients with HIBI with secondary brain hypoxia.

**Registration:**

URL: https://www.clinicaltrials.gov; Unique identifier: NCT03609333.

## Abstract 2

Supplemental Digital Content is available in the text.

**Meet the First Author, see p 513**

The mortality and morbidity of hypoxic ischemic brain injury (HIBI) following return of spontaneous circulation (ROSC) after cardiac arrest is devastating,^1–3^ with survivors experiencing a spectrum of outcomes encompassing vegetative states to significant long-term functional and neuropsychiatric disabilities.^3^ The cerebrovascular pathophysiology of HIBI is theorized as a two-hit model with both primary and secondary brain ischemic and/or hypoxic insults contributing to overall injury severity.^4^ Primary injury begins at the time of circulatory arrest with the immediate cessation of cerebral blood flow (CBF). Secondary injury takes place following resuscitation and restoration of innate cardiac output and CBF. During this phase, brief cerebral hyperemia is followed by a prolonged phase of depressed CBF, termed the no reflow period.^5–7^ During no-reflow, cerebral oligemia and a reduction in convective cerebral oxygen delivery (CDO_2_) may precipitate secondary brain hypoxia and cell death.

A cornerstone in the critical care management of patients with HIBI post-resuscitation is the optimization of convective CDO_2_ through augmentation of mean arterial pressure (MAP) along with maintenance of normocapnic ventilation^8–10^ and arterial oxygen content.^11,12^ Yet, recent randomized trials aimed at improving convective CDO_2_ through augmentation of MAP have failed to demonstrate biological or clinical efficacy.^13,14^ Further, despite goal directed resuscitation guided by neuromonitoring, patients with HIBI continue to experience a significant burden of brain hypoxia after ROSC^15^ and show physiological signatures consistent with increased diffusion barriers between the cerebral microvasculature and parenchyma.^16^ An improved understanding of the multiple mechanisms underlying secondary brain hypoxia in humans with HIBI is needed to facilitate the development of effective therapeutic strategies that extend beyond the augmentation of convective CDO_2_.

Preclinical animal models suggest blood-brain barrier (BBB) breakdown,^17,18^ perivascular edema,^19,20^ cellular metabolic perturbations,^18,21^ endothelial injury,^17,22^ oxidative stress,^6,18^ and inflammation^18,23^ may all be key factors in the pathophysiology of HIBI in humans. These factors all have the potential to intersect with—and impede oxygen transport at—multiple levels of the oxygen cascade to ultimately precipitate secondary brain hypoxia. If secondary brain hypoxia is of pathophysiologic significance in humans with HIBI and related ischemic brain disease states, injurious effects may be expected to arise within the neurovascular unit (NVU), which comprises the principal anatomic and functional interface between neuronal, astroglial, mural (smooth muscle cells and pericytes), and cerebrovascular endothelial cells.^24^ As function of the NVU determines the control of microvascular blood flow, nutrient exchange and cellular homeostasis of brain parenchyma,^24^ secondary hypoxic injury to the NVU (ie, in addition to the primary injury) would be devastating.^24^ Therefore, it is imperative to characterize the injury pattern of the NVU, investigate the impact of secondary brain hypoxia on the NVU, draw insights into the pathophysiology of secondary brain hypoxia, and determine the potential modifiability of brain hypoxia after ROSC in humans with HIBI.

To investigate the pathophysiology of the secondary hypoxic injury that follows a global cerebral ischemic insult, we tested the hypothesis that post–cardiac arrest patients exhibiting persistent secondary brain hypoxia would demonstrate elevated biomarkers of neuronal, astroglial, and endothelial injury^17,22^ as well as inflammation^18,25^ compared with patients without secondary brain hypoxia. We further hypothesized that secondary hypoxia would be related to impaired cerebrovascular-to-parenchymal diffusion of O_2_, and that this hypoxia would be reduced with hyperosmolar therapy. Utilizing arterial and jugular venous bulb blood sampling to isolate the cerebrovascular bed and determine transcerebral release of NVU injury markers, we demonstrate that persistent brain hypoxia following ROSC in humans is associated with de novo release of biomarkers related to neuronal and astroglial injury, namely, neurofilament-light (NF-L), NSE (neuron-specific enolase), total tau, and UCH-L1 (ubiquitin C-terminal hydrolase L1) as neuronal biomarkers and GFAP (glial fibrillary acidic protein) as an astrocyte biomarker. Importantly, secondary brain hypoxia was related to a cerebral proinflammatory response but not concurrent cerebral endothelial injury. Our findings of elevated markers of neuronal and astroglial injury, BBB breakdown, and systemic endothelial injury were recapitulated at a physiological level by a reduction in cerebral oxygen gradients (improved cerebrovascular-to-parenchymal O_2_ diffusion) and an improvement in the partial pressure of brain tissue oxygen (PbtO_2_) following hypertonic saline infusion.

## Methods

### Data Availability

The data that support the findings of this study are available from the corresponding author upon reasonable request. An expanded methods section is available in the Data Supplement.

### HIBI Patient Study

We conducted a prospective interventional study of invasive neuromonitoring in 18 patients with HIBI with paired sampling of arterial and jugular venous bulb blood to isolate the cerebrovascular bed and quantify transcerebral release of brain biomarkers (ie, arterial-to-venous [A-v] gradients; H16-0466). Fourteen patients in this cohort have been reported on in previous manuscripts^15,16^; however, the study aims, hypotheses, data analysis, and results for this article are original. Patients were enrolled between November 2016 and October 2019 in the Vancouver General Hospital intensive care unit (ICU). Enrollment for this study is no longer ongoing and the study is now complete (NCT03609333). Inclusion/exclusion criteria and patient management are detailed in the expanded methods. Written informed consent was provided by a legally authorized representative.

### Blood Sample Collection

Simultaneous arterial and jugular venous bulb gases were taken on patients every 4 to 6 hours for measurement of pH, arterial carbon dioxide tension, arterial oxygen tension, and bicarbonate concentration. Further, arterial and jugular venous blood were simultaneously collected into serum separator tubes (Becton & Dickinson, Vacutainer, 367986) within the first 24 hours of neuromonitoring (24 [13–40] hours post-arrest). These samples were set upright in the dark for 10 minutes and then centrifuged at 600*g* for 10 minutes, with the serum supernatant aliquoted into cryovials and immediately frozen in a −80 °C freezer where they were stored for later analysis. All biomarker samples collected in the first 24 hours were collected before the administration of hypertonic saline (see below). Further, samples were collected from patients on day 2 and 3 if they remained alive and within the ICU.

### Neurophysiologic Monitoring

Multimodal invasive neuromonitoring was undertaken via a dual lumen cranial access bolt (Integra Lifesciences, Plainsboro, NJ) positioned at the frontal hair line on the side of the nondominant hemisphere. Intraparenchymal intracranial pressure (Camino, Integra Lifesciences, Plainsboro, NJ) and brain tissue oxygenation (Licox, Integra Lifesciences, Plainsboro, NJ) catheters were inserted via the dual lumen bolt and positioned in the subcortical white matter. Noncontrast head computed tomography (Somatom Sensation 32 scanner, Siemens, Germany) was undertaken upon admission and then again 24 hours thereafter to confirm adequate placement of the neuromonitoring catheters (Figure I in the Data Supplement). A continuous oximetric jugular venous bulb saturation catheter (Pediasat, Edwards Lifesciences, CA) was inserted retrograde in the dominant jugular vein, identified by bedside ultrasonography. Confirmation of the positioning of the catheter at the ipsilateral mastoid process was confirmed with anterior—postural and lateral skull base x-ray film (Figure I in the Data Supplement). The median duration of neuromonitoring in our study was 43 hours (range, 12–164 hours). During this monitoring period, clinical directive was to maintain normoxemia (Pao_2_ 80–100 mmHg) and normocapnia (Paco_2_ 35–45 mmHg) for the duration of the study.

### Hypertonic Saline

Hyperosmolar therapy, using hypertonic saline infusion was administered as part of our institutional protocol for episodes of intracranial hypertension (nonstimulated intracranial pressure episode of >20 mm Hg for >10 minutes). A dose of 1 to 2 mL/kg of 5% hypertonic saline was administered over 15 minutes via a central venous catheter. Serum sodium was measured before and after 5% hypertonic saline administration.

### Healthy Controls Protocol

Fourteen healthy males (25±5 years of age; BMI of 24±3 kg/m^2^) were enrolled in a separate study, with unique research hypotheses, but are included here to provide normative control data for transcerebral A-v gradients of brain biomarkers (no data from this cohort has been previously published). This study was approved by the UBC Clinical Research Ethics Board (H18-01764) with the healthy volunteers providing written informed consent before participation.

Participants were instrumented with an internal jugular venous and radial arterial catheter. Participants had ≥20 minutes to rest following cannulation while the remaining experimental set-up for monitoring minute ventilation, measuring the partial pressures of end-tidal carbon dioxide (P_ET_CO_2_) and oxygen (P_ET_O_2_) was completed. Dynamic end-tidal forcing was utilized to hold P_ET_O_2_ and P_ET_CO_2_ steady^26^ for 10 minutes before and during blood sample collection. Arterial and jugular venous blood samples were simultaneously collected into serum separator tubes (367986, Becton & Dickinson, Vacutainer), set upright in the dark for 10 minutes and then centrifuged at 600*g* for 10 minutes (4 °C), with the serum supernatant aliquoted into cryovials and immediately snap-frozen in liquid N_2_ and then stored in a −80 °C freezer for later analysis.

### Biomarker Analysis

Serum samples from both healthy controls and patients with HIBI were analyzed for markers of neuronal and astroglial injury with the Quanterix platform using the Simoa HD-1 analyzer (Billerica, MA) by an investigator blinded to the patients’/controls’ physiological and outcome data. The following assays were preformed: GFAP discovery kit (102336), which reflects astroglial damage and BBB permeability^27^; ubiquitin carboxyl-terminal hydrolase L1 (UCH-L1) discovery kit (102343), which reflects neuronal cell body injury^27^; total tau advantage kit (101552) and NF-L advantage kit (103186), which reveal axonal damage; NSE discovery kit (102475), an intracytosolic enzyme, which represents neuron cell body injury.^27^

Inflammatory cytokines (ILs [interleukins] 6 and 10, and TNF-α (tumor necrosis factor-α) were quantified using the Simoa HD-1 platform from Quanterix (Billerica, MA) using a cytokine 3-plex A advantage assay (101160).

Markers of endothelial injury were measured as follows. E-selectin, P-selectin, sICAM-3 (soluble intracellular adhesion molecule 3), and thrombomodulin were analyzed using the MSD Human Vascular Injury 1 Kit (K15135C, Meso Scale Diagnostics, MD). Syndecan-1 (LSEHSDC1) and von Willebrand Factor (LSEHVWF) were quantified using Invitrogen Human Elisa Kits (Invitrogen, CA). Details for all biomarker analyses are provided in the expanded methods.

### Statistical Analysis

All statistical analyses were performed in GraphPad Prism (Version 9.1.2). Systemic hemodynamic physiology was variable within and between patients as noted in Figure II in the Data Supplement. Therefore, to make PbtO_2_ comparable relative to systemic hemodynamic conditions, we stratified by brain normoxia/hypoxia based upon PbtO_2_ values that were recorded when MAP was <100 mmHg. A PbtO_2_ value that occurred when MAP was >100 mmHg was not included in the 24-hour mean PbtO_2_ value utilized for determining patient stratification to account for removal of monitoring artifacts (eg, flushing of the arterial line) and to adhere to current treatment paradigms that do not involve MAP augmentation above 100 mmHg^13,14^ rendering these values of limited physiological or clinical relevance as it pertains to hypoxic brain injury.

This study included a sample of convenience. As this cohort of patients represents a first-in-human investigation of secondary brain hypoxia in the setting of HIBI post–cardiac arrest, no historical data were available for a priori power calculations. All data were assessed for normality using a Shapiro-Wilks test, with parametric and nonparametric tests used when appropriate (as described below). Data are presented as mean±SD or median (interquartile range) where appropriate.

For physiological data, the 6-hour binned neuromonitoring data (MAP, intracranial pressure, cerebral perfusion pressure [CPP], pressure reactivity index) were assessed using linear mixed effects models (patient as a random effect) with both time (0–6 versus 7–12 versus 13–18 versus 19–24 hours) and brain oxygenation (normoxia versus hypoxia) as fixed factors. Twenty-four hour mean data were compared using a Mann-Whitney *U* test.

Differences in arterial biomarker levels (brain biomarkers, cytokines, and endothelial markers) between patients with HIBI with brain normoxia and hypoxia as well as controls were made using a Kruskal-Wallis test. Following the detection of a significant effect, post hoc testing was conducted and corrected for multiple comparisons with a Dunn multiple comparisons test. The presence of A-v gradients for each biomarker was assessed using a 2-tailed single sample *t* test or Wilcoxin Signed Ranks test (population mean or median set at zero). Further, between group comparisons of A-v gradients were made using a Kruskal-Wallis test. Following the detection of a significant effect, post hoc testing was conducted and corrected for multiple comparisons with a Dunn multiple comparisons test.

To assess the influence of hypertonic saline on PbtO_2_ and related variables, the hypoxic patient data were compared pre-to-post hypertonic saline with a Wilcoxin Signed Ranks test. A *P* of <0.05 was considered statistically significant for all hypothesis testing.

It is acknowledged that this study includes a relatively small sample of participants, which may increase the risk of Type 2 error, but also includes repeated comparisons that may lend themselves to increased risk of Type 1 error. Therefore, in addition to significance testing individual data is displayed for the sake of transparency and improved ease of data interpretation.

## Results

Eighteen post–cardiac arrest patients with HIBI (6 female; 44±17 years of age; BMI of 25±6 kg/m^2^) admitted to Vancouver General Hospital (Vancouver, BC, Canada) that met the inclusion criteria were consecutively enrolled in this study between November 2016 and November 2019. All patients had >10 minutes from the time of cardiac arrest to ROSC and were enrolled within 72 hours following their arrest. Patient details are included in Table I in the Data Supplement. This study conformed to the standards put forth in the Declaration of Helsinki, received institutional approvals (H16-00466; H18-01764), and was registered on www.clinicaltrials.gov (NCT03609333). Written informed consent was provided by a legally authorized representative.

### Post–Cardiac Arrest HIBI Patients Exhibit Secondary Brain Hypoxia.

As part of clinical monitoring, a frontal dual access cranial bolt was placed in the mid-pupillary sagittal line on the side of the nondominant hemisphere (Figure IIA in the Data Supplement),^15^ through which 2 microcatheters were advanced into subcortical frontal lobe white matter to directly record PbtO_2_ and intracranial pressure. To investigate the pathophysiologic sequelae of secondary brain hypoxia in HIBI, patients were stratified into 2 groups based upon their mean PbtO_2_ over the first 24 hours of neuromonitoring: (1) those wherein clinical management achieved brain normoxia (mean PbtO_2_ ≥20 mm Hg) and (2) those with persistent brain hypoxia despite neuromonitoring-guided clinical management (mean PbtO_2_ <20 mm Hg; Figure IIB in the Data Supplement).^15,28,29^ Eight patients were classified as normoxic while 10 were classified as hypoxic.

The differences in PbtO_2_ between patients with HIBI with brain normoxia and hypoxia manifested despite no statistical differences in MAP over the first 24 hours of monitoring (88 [85–94] versus 87 [80–97] mm Hg over 24 hours; *P*=0.98; Figure IIC and IID in the Data Supplement). However, intracranial pressure was lower in the normoxic HIBI group (8 [7–13] versus 17 [13–26] mm Hg; *P*=0.0044; Figure IIE and IIF in the Data Supplement) resulting in a comparatively higher CPP relative to the patients with hypoxic HIBI (78 [74–85] versus 68 [60–76] mm Hg; *P*=0.016; Figure IIG and IIH in the Data Supplement). Average core body temperature (esophageal thermometer) over the first 24 hours of neuromonitoring was not different between the patients with brain normoxia (36.5 [34.25–37.0 °C]) compared with those with brain hypoxia (36.0 [35.0–36.0 °C]; *P*=0.669). There were no statistical differences in hemoglobin concentration (*P*=0.408), or Pao_2_ (*P*=0.372) over the first 24 hours of neuromonitoring. There was, however, a trivial difference in Sao_2_ between patients with HIBI with brain normoxia (96 [96–97] %) and brain hypoxia (96 [95–96] %; *P*=0.027). Paco_2_ was higher (and pH lower) in the patients with HIBI with brain hypoxia (39.5 [38.3–44.1] mm Hg) compared with brain normoxia (35.4 [31.7–38.3] mm Hg; *P*=0.019; Figure III in the Data Supplement).

Data pertaining to clinical and diagnostic markers of prognostication are reported in Table I in the Data Supplement. Specifically, loss of gray-white matter differentiation on head CT was noted in 1 patient in the normoxia group (12.5%, 1/8) and 7 patients (70%, 7/10) in the hypoxia group. Presence of restricted diffusion on diffusion weighted magnetic resonance imaging was noted in 2 patients in the normoxia group (33%, 2/6) and 4 patients in the hypoxia group (80%, 4/5). Clinical outcomes classified by the cerebral performance category in the normoxia and hypoxia groups are reported in Table I in the Data Supplement.

### Neuronal and Astroglial Injury Are Exacerbated in Post–Cardiac Arrest Patients With Secondary Brain Hypoxia

**Figure 1.**
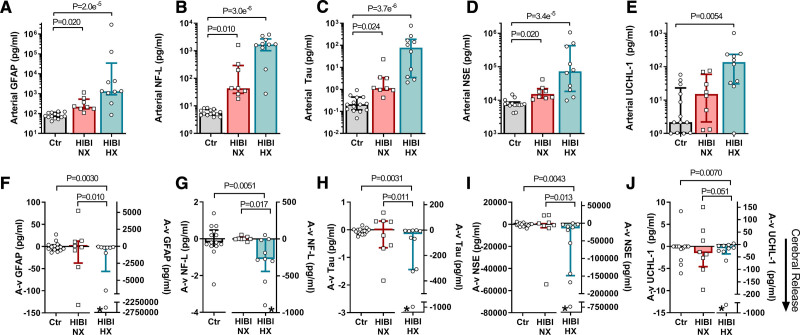
**Serum biomarkers of neurovascular unit and blood-brain barrier damage.** Arterial and jugular venous blood draws were acquired simultaneously for both healthy controls (Ctr) and for patients with hypoxic ischemic brain injury (HIBI) with brain normoxia (NX; red) and hypoxia (HX; cyan). Data are presented as bars (median±interquartile range) with individual data overlaid for each group. Arterial serum biomarker concentrations for GFAP (glial fibrillary acidic protein; **A**), neurofilament-light (NF-L; **B**), total tau (**C**), NSE (neuron-specific enolase; **D**), and ubiquitin carboxy-terminal hydrolase L1 (UCH-L1; **E**) are presented on the top row. For arterial serum GFAP, NF-L, Tau, and NSE, both HIBI groups had elevated biomarkers compared with controls, while only patients with HIBI with brain hypoxia had higher UCH-L1 than controls. Simultaneous collection of arterial and jugular venous blood, and calculation of the consequent arterial-to-venous gradient (A-v) allows for determination of biomarker release from the brain, where a negative (−) A-v gradient indicates cerebral release. These data for each biomarker are depicted on the second row in **F–J** (note differing *y* axis scales). Note that left and right y-axes are used in **F–J**. An asterisk (*) indicates cerebral release (ie, A-v gradient) is significantly different from zero as determined by a one sample Wilcoxon Signed Ranks test (or 1-sample *t* test when data were normally distributed). The cerebral release of each biomarker in the normoxic HIBI group was not different from that of controls, whereas the patients with hypoxic HIBI had a greater cerebral release than controls and patients with normoxic HIBI for each biomarker. Significance testing between groups was conducted with a Kruskal-Wallis test. Following the detection of a significant effect, post hoc testing was conducted and corrected for multiple comparisons with a Dunn multiple comparisons test. *P*<0.05 was considered statistically significant.

Given the observation that a large proportion of post–cardiac arrest HIBI patients exhibited persistent secondary brain hypoxia, we aimed to investigate the magnitude and pattern of NVU injury in patients with brain normoxia and hypoxia. Arterial and jugular venous blood samples were acquired from patients with HIBI in the first 24 hours of neuromonitoring (24 [13–40] hours post-arrest; the time of blood sample acquisition post-arrest did not differ between the patient groups, *P*=0.65), as well as from a healthy volunteer control group. Arterial serum levels of biomarkers related to neuronal and astroglial injury, GFAP (both *P*<0.05), NF-L (both *P*<0.05), total tau (both *P*<0.05), and NSE (both *P*<0.05) were elevated in both HIBI groups compared with controls, while UCH-L1 was only elevated in the HIBI group with brain hypoxia (*P*=0.0054) compared with controls (Figure 1A through 1E). These data indicate that simultaneous neuronal and astroglial injury (astrocytes, neural cell body, axonal) occurred in all the patients with HIBI.

Peripherally circulating biomarkers of NVU injury have been previously examined in a multitude of post–cardiac arrest studies,^30^ yet a key consideration is that arterial/peripheral venous serum levels for NVU injury biomarkers are impacted by factors other than injury severity such as the half-life of the biomarker, duration of the injury, and time of sampling relative to the time of arrest. Therefore, to provide greater insight into the magnitude and pattern of injury in patients with HIBI with brain normoxia and hypoxia, we examined arterial and jugular venous bulb blood samples to effectively isolate the cerebrovascular bed from the systemic circulation in an otherwise multisystem disease. The analysis of these transcerebral blood samples and calculation of the A-v gradient affords the ability to determine instantaneous transcerebral release of these biomarkers (a negative A-v gradient indicates cerebral release). We did not observe cerebral release of GFAP, NF-L, total tau, NSE or UCH-L1 in the control participants (Figure 1F through 1J). In the normoxic HIBI group, we did not observe cerebral release of GFAP (*P*=0.95), NF-L (*P*=0.74), total tau (*P*=0.74), NSE (*P*=0.10), or UCH-L1 (*P*=0.64), nor were their A-v gradients significantly different compared with controls. However, there was active cerebral release of GFAP (*P*=0.0078), NF-L (*P*=0.010), total tau (*P*=0.0039), NSE (*P*=0.0039), and UCH-L1 (*P*=0.0059) in the patients with HIBI with brain hypoxia (Figure 1F through 1J; Table II in the Data Supplement). The A-v gradients for GFAP, NF-L, total tau, NSE, and UCH-L1 were all greater by several orders of magnitude in the hypoxic HIBI group compared with the controls and the normoxic HIBI group (Figure 1F through 1J), indicating ongoing release of these biomarkers from the cerebral parenchyma. This indicates that at the time of sampling (24 [13–40] hours post-arrest) de novo neuronal and astroglial injury was occurring in the patients with HIBI with brain hypoxia but not the patients with HIBI with brain normoxia.

### Secondary Neuronal and Astroglial Injury Is Not Explained by Global Ischemic Burden

**Figure 2.**
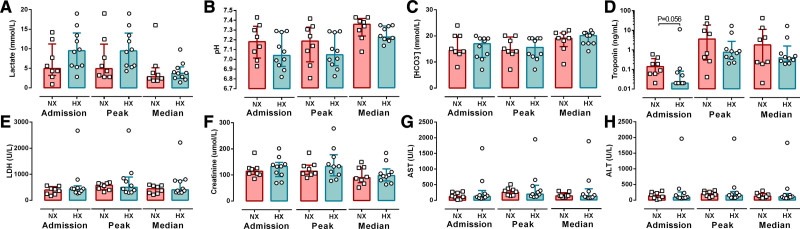
**Standard clinical laboratory values in cardiac arrest patients with brain normoxia and hypoxia.** The admission, peak, and median laboratory results are presented for lactate (**A**), pH (**B**), [HCO_3_^−^] (**C**), troponin (**D**), LDH (lactate dehydrogenase; **E**), creatinine (**F**), AST (aspartate aminotransferase; **G**), and ALT (alanine aminotransferase; **H**). Data are presented as bars (median±IQR) with individual patient data overlaid for both groups. Data were compared between normoxic (NX; red) and hypoxic (HX; cyan) patients with hypoxic ischemic brain injury at each time point using a Mann-Whitney *U* test. It is noted that there appears to be an outlier for LDH, AST, and ALT, with these values reflective of the same patient. Exclusion of this patient from the analysis does not alter the statistical output for these 3 variables (ie, no significant difference between groups).

Next we determined if standard clinical laboratory measures were also significantly different in the patients with HIBI with brain normoxia and hypoxia. As a part of routine clinical care, clinical laboratory measures were taken daily over the first 72 hours, allowing for characterization of the temporal evolution of clinical changes in normoxic and hypoxic HIBI patients. Over the first 72 hours of monitoring, the admission, peak, and median values for general markers of global ischemic injury including: lactate (*P*=0.15, *P*=0.14, *P*=0.59), pH (*P*=0.21, *P*=0.33, *P*=0.12), HCO_3_^−^ (*P*=0.65, *P*=0.69, *P*=0.84), and LDH (*P*=0.40, *P*=0.99, *P*=0.35) were not different between patients with HIBI with brain normoxia and hypoxia (Figure 2). Further, the admission, peak, and median cardiac troponin did not statistically differ between normoxic and hypoxic HIBI patients (*P*=0.056; *P*=0.062; *P*=0.99) nor did markers of hepatic and renal injury, creatinine, AST (aspartate aminotransferase), and ALT (alanine aminotransferase; Figure 2). Of note, the time to ROSC was not statistically different between the patients with HIBI with brain normoxia and hypoxia (16 [13–24] versus 18 [17–26] minutes; *P*=0.15; Figure IV in the Data Supplement). Moreover, the A-v gradients for markers of neuronal and astroglial injury were not correlated to time to ROSC (Table III in the Data Supplement). These results collectively suggest that the magnitude/severity of secondary injury in patients with HIBI is unrelated to the global ischemic burden of cardiac arrest.

**Figure 3.**
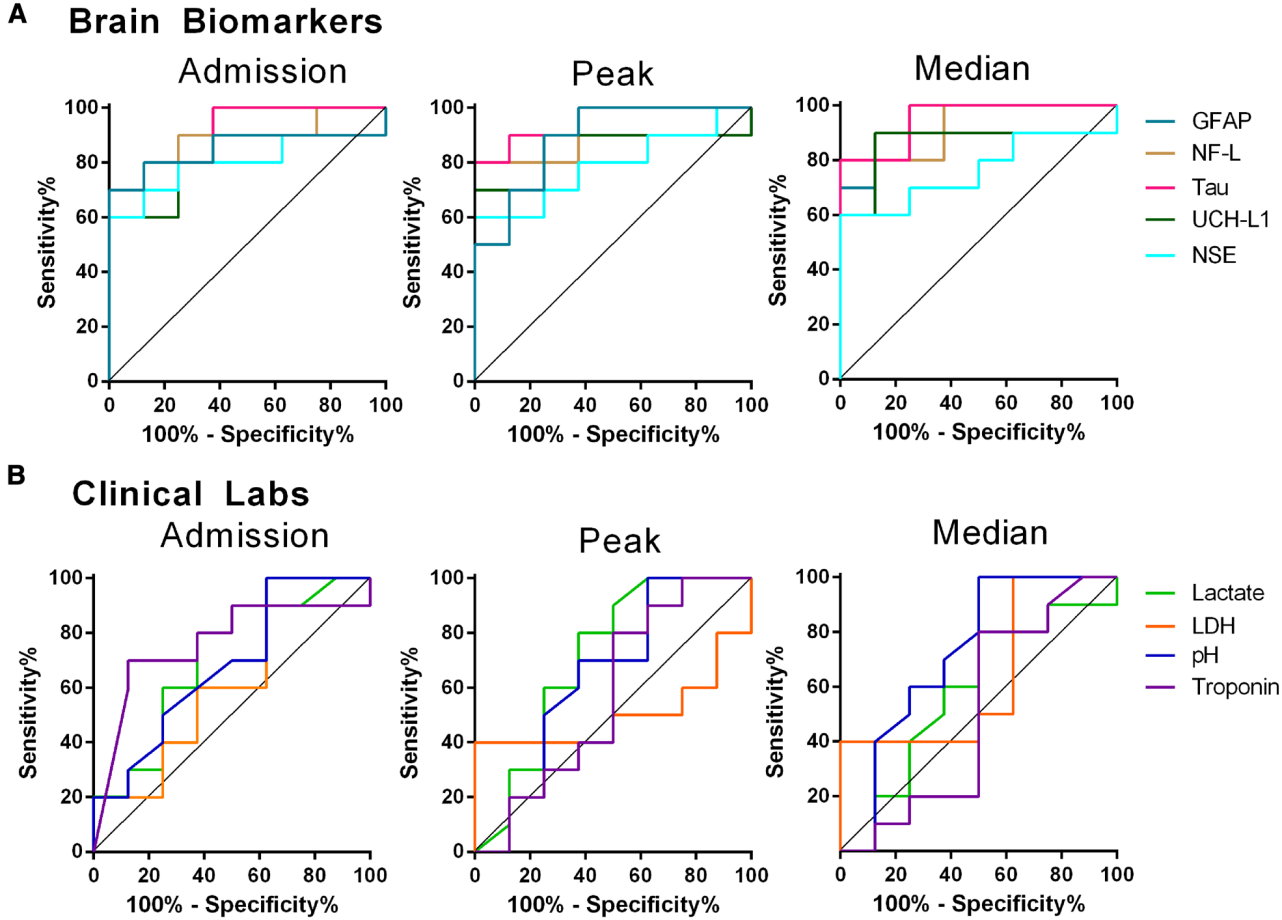
**Standard clinical laboratory values in cardiac arrest patients with brain normoxia and hypoxia.** Receiver operator curve characteristic analyses for admission, peak, and median brain biomarkers (**A**) and standard clinical laboratories (**B**) are depicted. All brain biomarkers were able to differentiate between brain normoxia and hypoxia with statistical significance (*P*<0.05 for all), while no clinical laboratory results were able to differentiate between brain normoxia and hypoxia. Specific data outputs for ROC analyses are in Table IV in the Data Supplement. GFAP indicates glial fibrillary acidic protein; LDH, lactate dehydrogenase; NF-L, neurofilament light chain; NSE, neuron-specific enolase; and UCH-L1, ubiquitin carboxy-terminal hydrolase L1.

Next, we evaluated the ability of neurological and basic clinical blood biomarkers to differentiate between patients with HIBI with brain normoxia and hypoxia using receiver operator characteristic curve analysis. The initial serum GFAP (AUC=0.85±0.10; *P*=0.013), NF-L (AUC=0.88±0.09; *P*=0.0077), total tau (AUC=0.90±0.07; *P*=0.0045), NSE (AUC=0.81±0.11; *P*=0.033), and UCH-L1 (AUC=0.80±0.11; *P*=0.026) levels were all able to accurately differentiate between brain normoxia and hypoxia, as were the peak and median levels of these biomarkers over the first 72 hours of neuromonitoring (Figure 3A; Table IV in the Data Supplement). Unsurprisingly, lactate, LDH, pH, troponin, and the other clinical laboratory results were not able to differentiate between these groups (Table IV in the Data Supplement).

### Secondary Brain Hypoxia Is Associated With a Cerebral Proinflammatory State, but Not Sustained Cerebral Endothelial or Glycocalyx Injury

**Figure 4.**
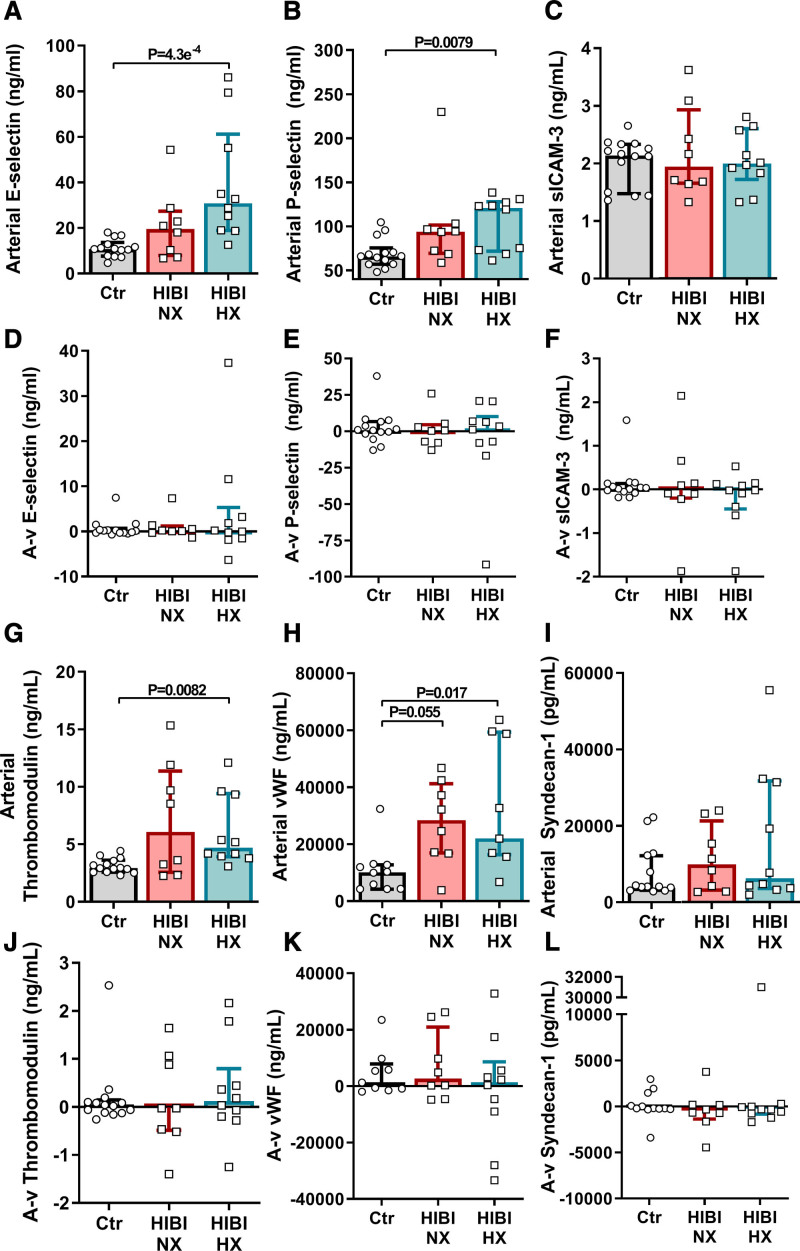
**Secondary brain hypoxia is not associated with sustained cerebral endothelial injury.** Arterial and jugular venous blood draws were acquired for both healthy controls (Ctr) and for patients with hypoxic ischemic brain injury (HIBI) with brain normoxia (NX; red) and hypoxia (HX; cyan). Data are presented as bars (median±interquartile range) with individual patient data overlaid for each group. Arterial serum biomarker concentrations are depicted for E-selectin (**A**), P-selection (**B**), sICAM-3 (soluble intracellular adhesion molecule-3; **C**), thrombomodulin (**G**), vWF (von Willebrand Factor; **H**) and syndecan-1 (**I**). Simultaneous collection of arterial and jugular venous blood, and calculation of the consequent arterial-to-venous gradient (A-v) allows for determination of biomarker release from the brain, where a negative (−) A-v gradient indicates biomarker release from the brain. These data for each biomarker are depicted under the corresponding arterial data within **D–F** and **J–L**. No significant cerebral release was detected. Significance testing between groups was conducted with a Kruskal-Wallis test. Following the detection of a significant effect, post hoc testing was conducted and corrected for multiple comparisons with a Dunn’s multiple comparisons test. *P*<0.05 was considered statistically significant.

As assessments of global ischemic injury failed to account for secondary brain hypoxia, we determined cerebral release (ie, A-v gradients) of markers of endothelial injury and inflammation to provide cerebrovascular-specific mechanistic insight (ie, independent of systemic ischemia-reperfusion) into the pathophysiology of HIBI. The patients with HIBI with brain hypoxia had elevated levels of arterial serum E-selectin (30.3 [18.7–60.4] ng/mL; Figure 4A; *P*=4.3×10^−4^), P-selectin (120.1 [71.6–127.1] ng/mL; Figure 4B; *P*=0.0079), thrombomodulin (4.7 [3.9–9.3] ng/mL; Figure 4G; *P*=0.0082), and von Willebrand Factor compared with controls (vWF; 21 629 [16 017–58 254] ng/mL; Figure 4H; *P*=0.017). No statistical differences in arterial sICAM-3 (Figure 4C) or syndecan-1 levels (Figure 4I) were found between any groups. We did not observe any significant cerebral release (ie, A-v gradients) for any of the measured biomarkers of endothelial injury (Figure 4D through 4F and 4J through 4L). Therefore, it appears there is an initial endothelial injury (presumably during cardiac arrest) in patients with HIBI, but the A-v gradients indicate that this injury is transient and does not continue unabated irrespective of the presence of persistent secondary brain hypoxia.

**Figure 5.**
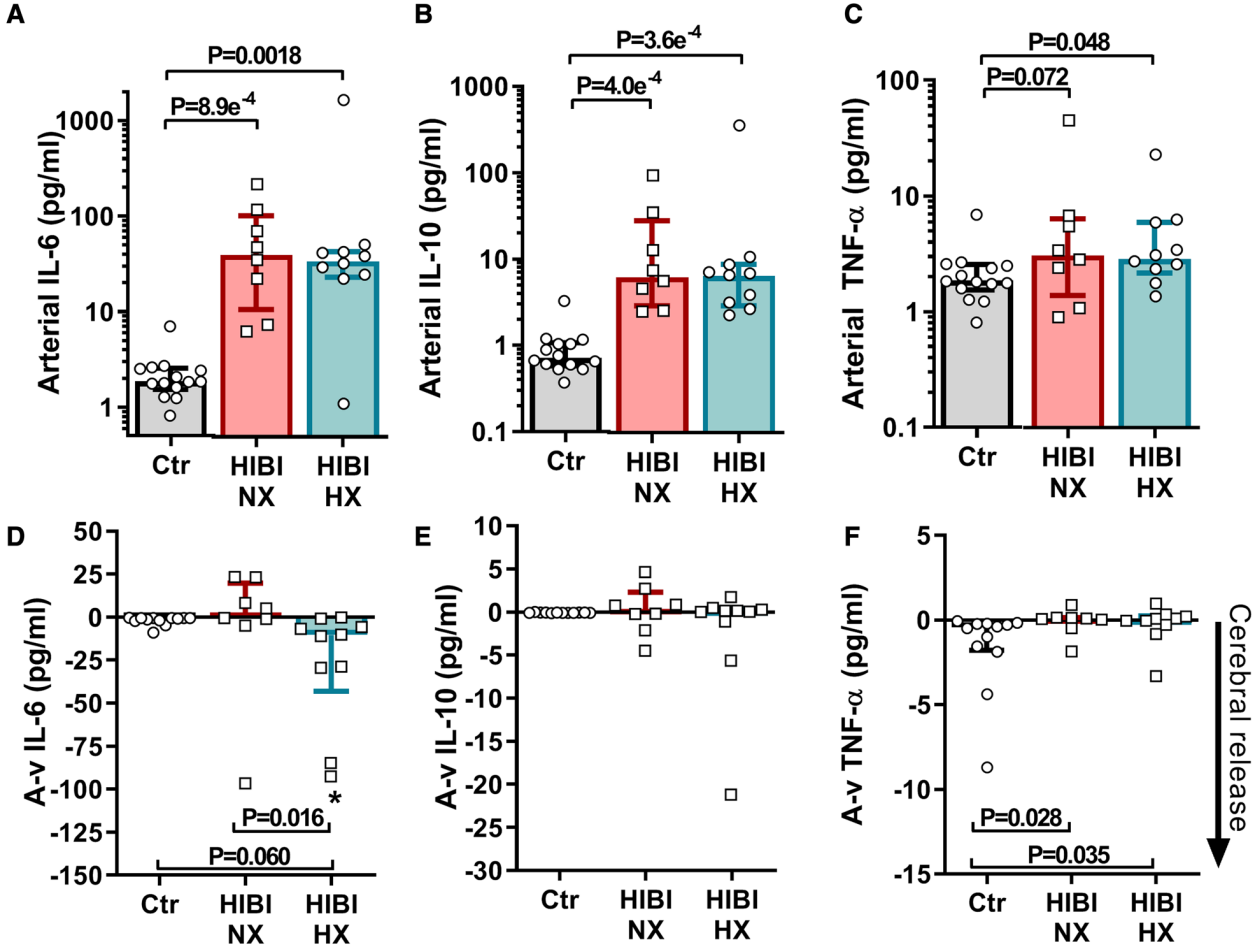
**Cerebral inflammation is associated with secondary brain hypoxia.** Arterial and jugular venous blood draws were acquired for both healthy controls (Ctr) and for patients with hypoxic ischemic brain injury (HIBI) with brain normoxia (NX; red) and hypoxia (HX; cyan). Data are presented as bars (median±IQR) with individual data overlaid for each group. Arterial serum biomarker concentrations for IL-6 (interleukin-6; **A**), interleukin-10 (IL-10; **B**) and TNF-α (tumor necrosis factor alpha; **C**) are presented on the top row. For arterial serum IL-6 and IL-10, both HIBI groups had elevated levels compared with controls, while only patients with HIBI with brain hypoxia had higher arterial TNF-α levels than controls. Simultaneous collection of arterial and jugular venous blood, and calculation of the consequent arterial-to-venous gradient (A-v) allows for determination of biomarker uptake/release from the brain, where a negative (−) A-v gradient indicates biomarker release from the brain. These data for each biomarker are depicted on the second row in **D–F**. The cerebral release for IL-6 in the normoxic HIBI group was not different from that of controls, whereas the patients with hypoxic HIBI had a greater cerebral release than controls and patients with normoxic HIBI. The cerebral release of IL-10 did not differ between any group while there was a trivial difference between the TNF-α A-v gradient in the control group and both HIBI groups. Significance testing between groups was conducted with a Kruskal-Wallis test. Following the detection of a significant effect, post hoc testing was conducted and corrected for multiple comparisons with a Dunn’s multiple comparisons test. An asterisk (*) indicates that an A-v gradient (cerebral release) is significantly different from zero. *P*<0.05 was considered statistically significant.

ILs 6 and 10 were elevated in the arterial blood of both the normoxic (*P*<0.05) and hypoxic (*P*<0.05) HIBI patients compared with controls (Figure 5A and 5B). There was a significant cerebral release of IL-6 in the hypoxic HIBI patients (10.3 [43.0–4.2] pg/mL; *P*=0.0039), which was greater compared with patients with normoxic HIBI and controls, who did not exhibit any significant cerebral release (ie, A-v gradients; Figure 5D). There was no significant cerebral release of IL-10 or TNF-α in any group (Figure 5E and 5F), albeit trivial differences for TNF-α A-v gradients existed between the controls and each HIBI group (Figure 5F). Collectively, this indicates cerebral-specific IL-6 release and associated neuroinflammation in the patients with HIBI with brain hypoxia.

### Hyperosmolar Therapy Mitigates Secondary Brain Hypoxia

**Figure 6.**
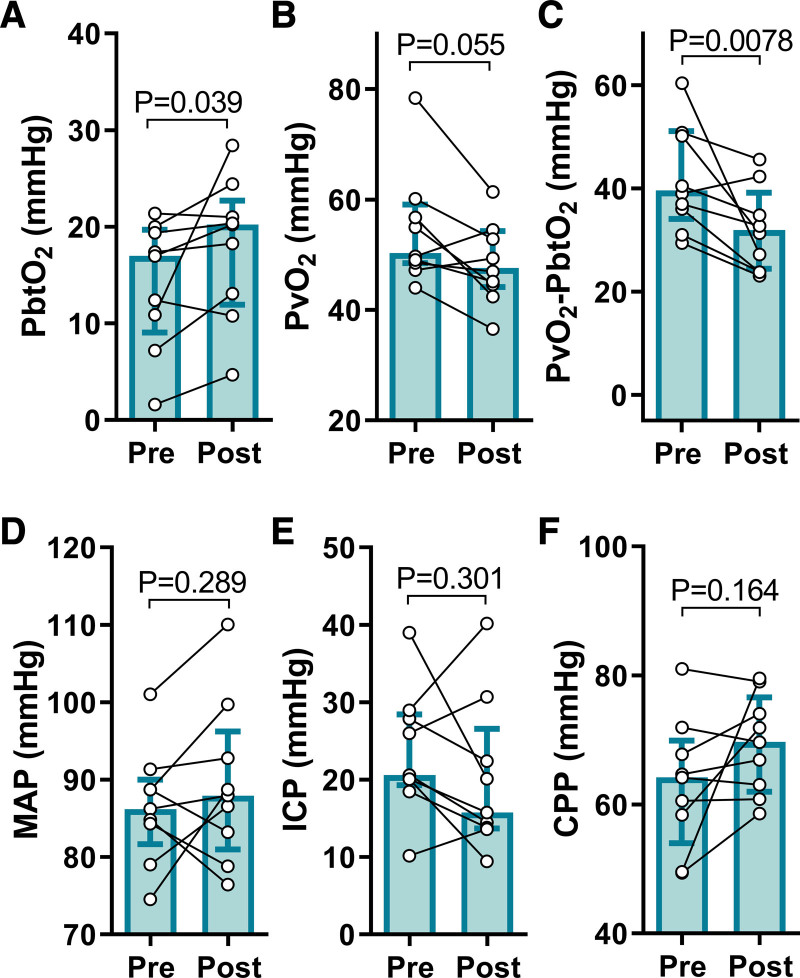
**Hypertonic saline increases brain tissue oxygen tension in post–cardiac arrest patients.** Patients with hypoxic ischemic brain injury (HIBI) exhibiting brain hypoxia (HX, n=9) are denoted by the cyan coloured bars (median±interquartile range). Individual patient data are overlaid. The 1-h mean immediately pre hypertonic saline infusion (Pre) and the 6-h mean immediately following infusion (Post) are depicted for brain tissue Po_2_ (PbtO_2_; **A**), partial pressure of jugular bulb venous oxygen (PvO_2_; **B**), the PvO_2_-PbtO_2_ gradient (**C**), mean arterial pressure (MAP; **D**), intracranial pressure (ICP; **E**), and cerebral perfusion pressure (CPP; **F**). Hypertonic saline infusion increased PbtO_2_ (*P*=0.039) and decreased the PvO_2_-PbtO_2_ gradient (*P*=0.0078) in the patients with hypoxic HIBI. These changes occurred despite no alteration in the systemic hemodynamic input to the brain as indicated by unaltered MAP (*P*=0.289), ICP (*P*=0.301), and CPP (*P*=0.164). Only 4 patients with normoxic HIBI received hypertonic saline based on clinical indication, thus while the data are presented for transparency in the supplement (Figure V in the Data Supplement), no statistical analyses were completed on these data. The hypoxic HIBI patient data were compared preto-post hypertonic saline with a Wilcoxon Signed Ranks test, with significance assumed at *P*<0.05.

Emerging evidence suggests that the pathophysiology of secondary brain hypoxia is in part attributable to impeded oxygen diffusion from the cerebral microvasculature into the parenchyma. Indeed, in human Patients with HIBI, PbtO_2_ does not always increase consequent to increased cerebral perfusion (ie, convective CDO_2_).^16,31^ To test this hypothesis, we assessed PbtO_2_ and cerebrovascular-to-parenchymal oxygen gradients before and following osmotherapy with hypertonic saline in 9 hypoxic (Figure 6) and 4 normoxic (Figure V in the Data Supplement) Patients with HIBI. It is noteworthy that these sample sizes are reduced because hypertonic saline was only administered when clinically indicated and as such, statistical analyses were not performed in the normoxic patients receiving hypertonic saline. The hypertonic saline infusion increased PbtO_2_ in the Patients with HIBI with brain hypoxia (17.0 [9.1–19.7] versus 20.2 [11.9–22.7] mm Hg; *P*=0.039; Figure 6A) and reduced the PvO_2_-PbtO_2_ gradient (39.6 [34.1–51.1] versus 32.0 [24.5–39.2] mm Hg; *P*=0.0078; Figure 6C), albeit PvO_2_ did not differ statistically. These improvements in PbtO_2_ occurred despite an unaltered systemic hemodynamic input into the brain, as indicated by the stable MAP (86.2 [81.7–90.0] versus 87.9 [81.0–96.2] mm Hg; *P*=0.29; Figure 6D), intracranial pressure (20.6 [19.3–28.5] versus 15.8 [13.7–26.6] mm Hg; *P*=0.30; Figure 6E) and CPP (64.2 [54.0–69.9] versus 69.7 [62.0–76.6] mm Hg; *P*=0.16; Figure 6F). Therefore, improved brain oxygenation and reduced O_2_ gradients may have manifested as a result of improved diffusion rather than perfusion, implicating cerebral edema in the pathophysiology of secondary brain hypoxia post–cardiac arrest.

## Discussion

We characterized injury to the NVU following cardiac arrest in human patients with HIBI with ongoing secondary brain hypoxia versus normoxia as well as in healthy controls. In these respective groups, we examined the effect of brain hypoxia on patterns of NVU injury by assessing transcerebral A-v gradients of neuronal, astroglial, and cerebrovascular endothelial injury biomarkers as well as inflammatory cytokines. Moreover, we demonstrated that persistent secondary brain hypoxia may be attenuated by resolving potential impairments in diffusive cerebrovascular-to-parenchymal O_2_ transport. Specifically, the present study demonstrates (1) persistent secondary brain hypoxia following resuscitation is associated with de novo release of biomarkers that reflect injury to neuronal and astroglial components of the NVU; (2) the presence of secondary brain hypoxia and associated neuronal and astroglial injury is unrelated to the global systemic burden of ischemia or endothelial injury produced by cardiac arrest; (3) secondary brain hypoxia associated neuronal and astroglial injury coincided with cerebral release of IL-6; (4) secondary brain hypoxia post–cardiac arrest is attenuated by hypertonic saline infusion indicating a potential role for cerebral edema, specifically in the perivascular distribution, and consequent diffusion limitation of oxygen transport into the cerebral parenchyma as a key pathophysiologic characteristic of HIBI.

**Figure 7.**
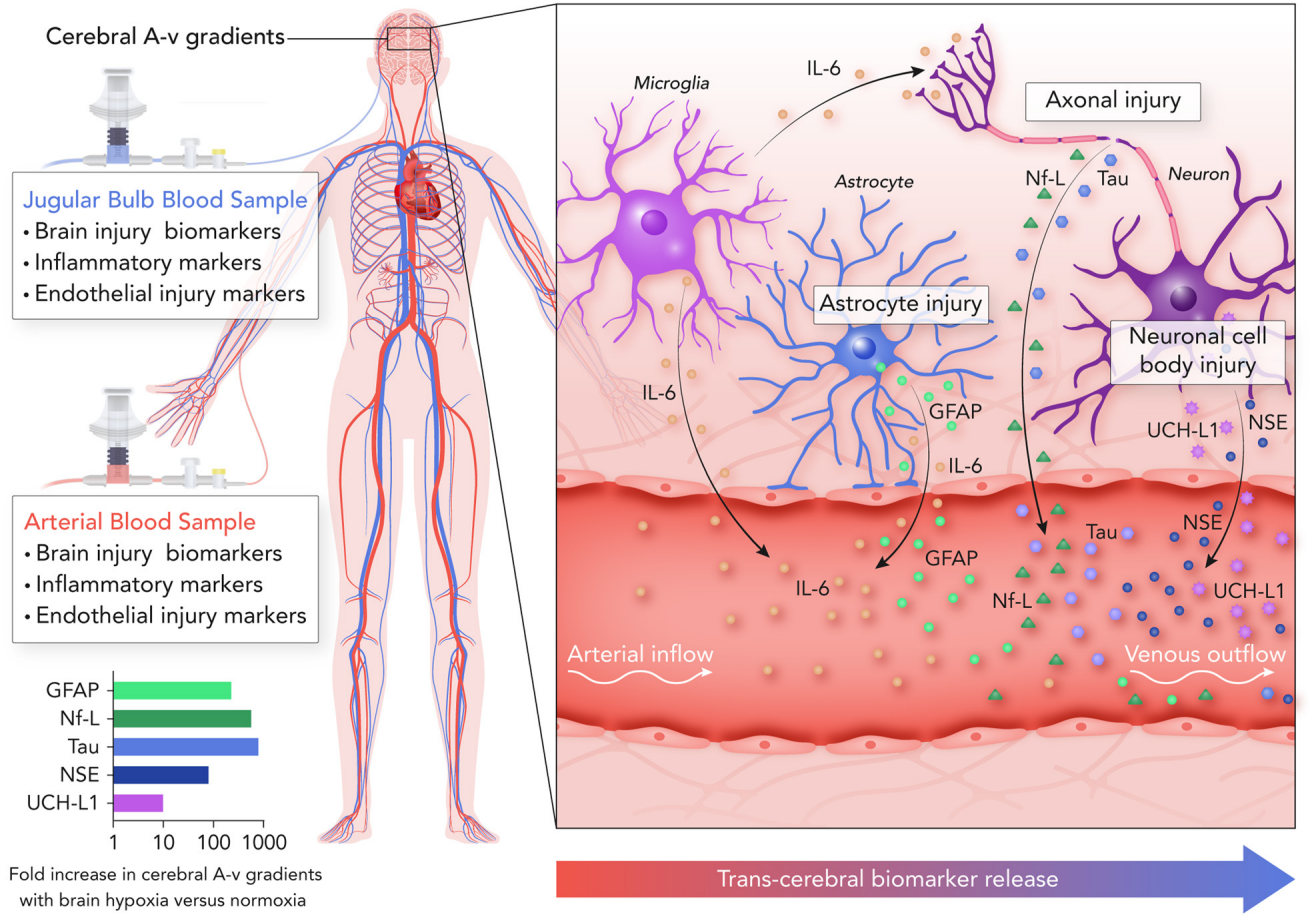
**Assessing secondary brain injury in post–cardiac arrest patients with hypoxic-ischemic brain injury through transcerebral biomarker release.** This schematic outlines the measurement of serum biomarkers of brain injury in arterial and jugular venous blood samples to quantify transcerebral biomarker release (ie, the cerebral arterial-to-venous [A-v] gradient). This allows for experimental isolation of the cerebrovascular bed in the setting of an otherwise multisystem injury. The present investigation observed differences in the cerebral A-v gradients of markers of neuronal cell body (NSE [neuron-specific enolase] and UCH-L1 [ubiquitin carboxyl-terminal hydrolase L1]), axonal (Nf-L & Tau), and astroglial (GFAP [glial fibrillary acidic protein]) injury as well as neuroinflammation (IL-6 [interleukin-6]) between patients with secondary brain hypoxia compared with normoxia following a cardiac arrest. The difference in the magnitude of cerebral A-v gradients between patients with secondary brain hypoxia vs normoxia is demonstrated in the graph on the bottom left, with an accompanying illustration highlighting the sites of injury within the neurovascular unit on the right side of the figure.

There is a long history of measuring markers of NVU injury to quantify the severity of HIBI following cardiac arrest^32^ given that neurological injury is the primary cause of death in those that survive to hospital admission.^1,2^ Yet, measurement of one marker, as is typical, does not fully characterize the injury that may be taking place across the entire NVU. Herein, we demonstrate that de novo transcerebral release of NVU specific biomarkers reflecting astroglial injury (GFAP),^33^ neuronal cell body injury (NSE, UCH-L1),^34,35^ and axonal injury (NF-L, tau) occurs simultaneously in the context of post-ROSC brain hypoxia (Figure 7). The unique measurement of cerebrovascular A-v gradients in the present study allowed for isolation of the cerebrovascular bed and quantification of instantaneous and cerebral-specific injury, which demonstrated that neuronal and astroglial injury was only actively occurring in patients with HIBI with persistent secondary brain hypoxia (hypoxic HIBI group), but not in the normoxic patients with HIBI wherein brain normoxia had been restored following resuscitation (no statistical difference from healthy controls). Therefore, post–cardiac arrest patients with HIBI are susceptible to ongoing tissue injury with persistent secondary brain hypoxia as indicated by the active release of biomarkers of neuronal and astroglial injury despite the implementation of neuromonitoring-guided goal directed care (involving factors such as MAP augmentation).^15^ This observation may explain the lack of efficacy of MAP augmentation recently demonstrated in post–cardiac arrest clinical trials^13,14^ and necessitates a better understanding of the pathophysiological mechanisms that underlie secondary brain hypoxia associated NVU injury following cardiac arrest in humans.

The presence of secondary brain hypoxia and associated neuronal and astroglial injury following cardiac arrest was not related to measures of overall ischemic burden as indexes of global ischemic injury (eg, lactate & pH) and time to ROSC (Figure IV and Table III in the Data Supplement) were unrelated to the presence of secondary brain hypoxia or the magnitude of neuronal and astroglial injury. However, it has been well documented in other critical illnesses (eg, sepsis and traumatic brain injury)^36,37^ and more recently in post–cardiac arrest patients^25,38^ that systemic circulating markers of inflammation and endothelial injury are related to poor outcomes. Indeed, post-ROSC ischemia-reperfusion injury incites a proinflammatory state thought to underlie endothelial injury and BBB breakdown in human patients with HIBI,^25^ an observation that is supported by preclinical experiments.^18,22^ Such endothelial damage and consequent BBB breakdown precipitates cerebral edema,^17,22,39^ which will impose a diffusional barrier to oxygen transport within the cerebral parenchyma.^40^

Endothelial injury was evident in the Patients with HIBI. That some arterial markers of endothelial injury were elevated but no A-v gradients were present may be taken to reflect an initial ischemia-reperfusion injury to the endothelium^41^ that does not persist thereafter (at least relative to the brain). Thus, while cerebral endothelial injury may be an important early factor in the pathophysiology of HIBI, it does not seem that ongoing cerebral endothelial injury is related to secondary brain hypoxia.

Cerebral inflammation leads to increased cerebral IL-6 production.^23,42,43^ We demonstrate increases in circulating systemic IL-6^25^ but not TNF-α in agreement with previous work in mice.^18^ However, we also provide nuanced insight into cerebral-specific inflammation by demonstrating that persistent secondary brain hypoxia was related to cerebral release of IL-6. This is in agreement with preclinical work demonstrating increases in hippocampal IL-6 following cardiac arrest.^44^ IL-6 is produced by astrocytes (and other cells) in response to inflammation^42^ and may represent a key pathophysiological process of secondary hypoxic brain injury in humans with HIBI given the marked injury to astrocytes evidenced by the cerebral release of GFAP in the HIBI group with brain hypoxia. Our data therefore suggest that astrocyte IL-6 production may represent a potential therapeutic target^45,46^ as reducing cerebral IL-6 production is associated with reduced apoptotic signaling following cardiac arrest.^44^ To this effect, clinical trials assessing the influence of IL-6 receptor blockade with Tocilizumab on outcome following cardiac arrest are ongoing (NCT03863015). It is also possible that cerebral production of, and A-v gradient for, IL-6 may be in part explained by microglial activation and/or the expression of adhesion molecules (eg, E-selectin) within the cerebral vasculature and consequent macrophage infiltration and cytokine release.^23^

An emerging hypothesis is that secondary brain hypoxia is related to a diffusion limitation that impedes cerebrovascular-to-parenchymal oxygen transport,^16,31^ which renders MAP augmentation less effective for ameliorating brain hypoxia in these vulnerable patients. The demonstration of cerebral release of GFAP in the hypoxic patients with HIBI, along with cerebral release of IL-6 and elevated arterial markers of endothelial injury, all suggest BBB disruption,^17^ fluid extravasation,^17^ and consequent perivascular edema^40^ as a potential mechanism for diffusion limitation and persistent secondary brain hypoxia in humans with HIBI. We extend this notion by experimentally demonstrating that hypertonic saline infusion improved PbtO_2_ and reduced the PvO_2_-PbtO_2_ gradient, which signifies improved oxygen transport into the cerebral parenchyma (ie, likely an improvement in cerebrovascular-to-parenchymal diffusion).^16,40^ Previous work has demonstrated an improvement in CBF with hypertonic saline infusion.^47–49^ However, while we cannot account for changes in cardiac output and any consequent influence on CBF^50^ following hypertonic saline infusion, the unaltered CPP in the current study lends support to the notion that the improvements in PbtO_2_ we observed were secondary to improved diffusion and not perfusion, likely a result of decreased perivascular edema.^19,20,40^ Therefore, these data suggest that therapeutic targeting of diffusion limitation may hold efficacy for mitigating secondary brain hypoxia in instances where MAP augmentation is not effective.

Assessment of human patients within the clinical setting precludes the experimental designs and uniformity that are typical of preclinical models of HIBI, where, for example, arrest times are highly controlled.^6^ However, such variability is integral to produce a range in the severity of brain hypoxia, a prerequisite to derive the important pathophysiologic relationships described herein. Previous efforts to elucidate the mechanisms of HIBI post–cardiac arrest and key contributors to mortality have not been capable of disentangling systemic physiology and ischemic injury from that of cerebral-specific injury. Our quantification of cerebral A-v gradients circumvents the potential for conflating systemic and cerebral injuries by isolating changes in multiple injury markers (neuronal, astroglial, endothelial, etc) to the cerebral vasculature/parenchyma (Figure 7). This has allowed us to demonstrate that a unique injury pattern occurs in patients with a PbtO_2_ <20 mmHg (ie, hypoxia) versus those with a PbtO_2_ >20 mmHg (ie, normoxic) post-resuscitation. Indeed, this provides evidence that 20 mmHg may be a meaningful cutoff for brain hypoxia in post–cardiac arrest patients, a notion that was previously extrapolated from traumatic brain injury literature.^15,28^ While the purpose of this study was not to assess differences in clinical outcomes between groups due to the small sample size, it is noteworthy that in the group with brain normoxia, 6 patients had a favorable outcome (CPC1-2), representing 75% of the normoxic patients, whereas in the patients with brain normoxia, only 2 patients (20%) had a favorable outcome (Table I in the Data Supplement).

While we have assessed multiple aspects of the NVU as it relates to secondary hypoxic injury in humans, it is important to address that other factors not examined herein cannot be disregarded, such as the potential for pericyte injury^51^ and the potential contributions of microglia and vessel-associated macrophages.^23^ This study comprises a limited sample of human patients; yet, this limitation is offset by the state-of-the-art invasive and molecular methodology employed; and, for the first time, coupling of our HIBI patient data with that of a healthy control group that also underwent invasive catheterization to determine normative cerebral A-v gradients. We recognize that the generalizability of our findings may be limited due to the noncardiac causes of arrest in our cohort and our findings may not be directly relatable to patients with HIBI with sudden cardiac arrest resulting from either a primary arrhythmia or antecedent acute coronary syndrome. Given the nature of our exclusion criteria, we were unable to include patients with concomitant acute coronary syndromes because of contraindications to the insertion of invasive neuromonitoring in the setting of administration of anticoagulants or antiplatelet therapy. Additionally, the age of our cohort was younger than a typical post cardiac arrest HIBI population and although this may be viewed as a limitation, it should be noted that the strengths of our findings are enhanced as direct control comparisons may be made based upon the similar ages of our HIBI cohort and health controls.

Finally, it is important to note that we did not directly measure CBF, the regulation of which is multifaceted.^52^ Therefore, while we have used CPP as a surrogate for CBF, the regulation of CBF involves many factors in addition to perfusion pressure such as neuronal activity, arterial blood gases, and temperature. As these factors were not markedly altered during the stable periods of neuromonitoring (eg, constant propofol sedation, relatively steady arterial blood gases) it is likely that CPP provided a reasonably accurate (albeit not precise) proxy for CBF. Future work utilizing direct intraparenchymal measures of CBF are needed to delineate the importance of diffusion limitations to O_2_ transport in the pathophysiology of post–cardiac arrest HIBI. Nonetheless, small differences in CBF per group could not alone account for the marked differences in cerebral release of biomarkers we observed where the release of GFAP (230 fold greater), NF-L (577 fold greater), Tau (800 fold greater), NSE (81 fold greater), and UCH-L1 (10 fold greater) were much greater in magnitude than what could be accounted for by even large differences in CBF between patients with brain hypoxia compared with normoxia (Figure 7).

In conclusion, we demonstrate that persistent secondary brain hypoxia is associated with de novo injury across neuronal and astroglial domains of the NVU. Further, secondary brain hypoxia was related to a cerebral proinflammatory response but not concurrent cerebral endothelial injury. These biomarker findings were recapitulated at a physiological level by an apparent mitigation of diffusion limitation, improved O_2_ transport, and an improvement in PbtO_2_ following hyperosmolar therapy with hypertonic saline infusion.

## Acknowledgments

We acknowledge the staff of Vancouver General Hospital intensive care unit and our colleagues at Addenbrooke’s Hospital, University of Cambridge for their support.

## Sources of Funding

This study was funded by operating grants from the Canadian Institute of Health Research (No. 437644), Laerdal Foundation, Heart and Stroke Foundation of Canada and Vancouver General Hospital Foundation. M.S. Sekhon is funded through the Clinician Scientist Award from Vancouver Coastal Health Research Institute and a Health-Professional Investigator Award from the Michael Smith Foundation. D.E. Griesdale is funded through a Health-Professional Investigator Award from the Michael Smith Foundation for Health Research. R.L. Hoiland is funded by a Michael Smith Foundation for Health Research Trainee Fellowship, UBC Bluma Tischler Post-Doctoral Fellowship, and the Darin Daniel Green Memorial Scholarship.

## Disclosures

None.

## Supplemental Materials

Expanded Materials and Methods

Data Supplement Tables I–IV

Data Supplement Figures I–V

## Supplementary Material



## Novelty and Significance

### What Is Known?

- Cardiac arrest patients suffer from a primary neurological injury during arrest as well as secondary neurological injury in the acute care setting following resuscitation.
- Hypoxic ischemic brain injury remains the predominant cause of adverse outcome after cardiac arrest and the prevention of secondary injury following resuscitation is a mainstay in patient management.
- Following return of spontaneous circulation, brain hypoxia is known to be prevalent; however, the impact on the neural, glial, and endothelial cells that comprise the neurovascular unit is unknown.
