# Supplementary material for: Brain Hypoxia Is Associated With Neuroglial Injury in Humans Post–Cardiac Arrest
Source: Circ Res. 2021 Jul 21;129(5):583–97. doi: 10.1161/CIRCRESAHA.121.319157 (PMC8376277; doi:10.1161/CIRCRESAHA.121.319157)
Supplement: Supplementary file 1 [file res-129-583-s001.pdf]

SUPPLEMENTAL MATERIAL

DETAILED METHODS

Study Participant Eligibility Criteria

Eligible patients met the following inclusion criteria: a) age ≥ 19; b) sustained cardiac arrest > 10 minutes requiring cardiopulmonary resuscitation; c) post-return of spontaneous circulation (ROSC) Glasgow Coma Score ≤ 8 and motor score ≤ 5 in the absence of clinical confounders (sedative medications, core body temperature < 35°C, electrolyte disturbances, hypoglycemia); d) study enrollment with 72 hours of the initial cardiac arrest. Patients were excluded if they met any of the following: a) anticipated withdrawal of life-sustaining therapies within the next 24 hours; b) previous or current traumatic brain injury, intracranial hemorrhage or stroke; c) concurrent coagulopathy (international normalized ratio > 1.5, prothrombin time > 40 seconds, platelet count < 100 x 10^{9} cells); d) concurrent anti-platelet or anticoagulant medications; e) anticipated cardiac catheterization within the next 7 days.

Patient Management

Patients were managed according to our institutional post-cardiac arrest management protocol, which is in keeping with international guideline recommendations. Specifically, patients were managed with targeted temperature management (35 to 36°C) for 24 to 48 hours using intravascular cooling (Solex®, Zoll, USA). The primary sedative was intravenous propofol (0 to 80µg/kg/min) and second line agents included intravenous midazolam (0 to 30mg/h) or narcotics (fentanyl 0 to 200mcg/h). Propofol was kept constant across the neuromonitoring period. Clinical directive was to maintain normoxemia (PaO_2 80 to 100mmHg) and normocapnia ($PaCO_2$ 35 to

45mmHg) for the duration of the study. Mean arterial pressure > 65mmHg was targeted at the discretion of the attending physician with intravenous norepinephrine as the first line vasopressor. Finally, hemoglobin concentration was maintained > 90g/L. The neuromonitoring data was available for the attending physician to view and use in the post-resuscitation management of the patients in keeping with our institution's neuromonitoring guidelines.

Hypertonic Saline

In the normoxic patients, hypertonic saline was infused 32±33 hours following the insertion of neuromonitoring. In the hypoxic patients, hypertonic saline was infused 12±11 hours following the insertion of neuromonitoring. Serum sodium concentration increased from 142.6±3.3 prior to infusion to 146.4±4.4 mmol/L 6 hours following the infusion ($P<0.001$).

Healthy controls Normoxia and Hypoxia Catheterization

Upon arrival to the laboratory, participants voided their bladder and assumed the supine position for instrumentation with an internal jugular venous and radial arterial catheter. Using sterile technique, a 20G arterial catheter (Arrow®, Markham, ON, Canada) was advanced into the left radial artery under local anesthesia (Lidocaine, 1.0%). This technique was assisted via the use of ultrasound guidance. Subsequently, a 13G central venous catheter (Cook Medical®, Bloomington, IN, USA) was advanced into the right internal jugular vein, again under sterile conditions and with the use of local anesthesia and ultrasound guidance. The catheter was then advanced up to 15 cm cephalad.[53] This technique has been previously demonstrated by our group to lead to catheter tip placement in the jugular bulb, which is importantly proximal to the facial

vein.[54] Further, correct placement was additionally determined by participants noting a sensation in their ear upon full insertion of the catheter.[53] Fulfilment of these techniques leads to ≤3% contamination by extra-cerebral blood.[53]

Biomarker Analysis

Serum samples from both healthy controls and HIBI patients were analyzed for markers of neuronal and astroglial injury with the Quanterix® platform using the Simoa HD-1® analyzer (Billerica, MA, USA) by an investigator blinded to the patients' physiological and outcome data. The following assays were preformed using the manufacturers protocols and diluted on board at a 4-fold dilution and run in duplicate; glial fibrillary acidic protein (GFAP) discovery kit (102336), which reflects astroglial damage and BBB permeability;[27] ubiquitin carboxyl-terminal hydrolase L1 (UCH-L1) discovery kit (102343), which reflects neuronal cell body injury;[27] total tau advantage kit (101552) and neurofilament-light (NF-L) advantage kit (103186), which reveal axonal damage. Neuron specific enolase (NSE) discovery kit (102475), an intra-cytosolic enzyme, which represents neuron cell body injury[27], was run using the manufacturers protocols with samples diluted off board at a 50-fold dilution and run in duplicate. For each assay, subjects were randomized into three runs, each of which included an 8-point standard curve, two manufacturer provided controls and three internally provided serum controls. For markers that had serum samples above the upper limit of detection, these samples were re-analyzed using a higher off-board dilution than the standard outlined in the manufactures protocol. These adaptions were as follows: GFAP was diluted up to either a 200 or 1000-fold dilution, NF-L was run at a 20-fold dilution, and NSE was diluted up to either a 400 or 1000-fold dilution.

Inflammatory cytokines (IL-6, IL-10, & TNF-α) were quantified using the Simoa HD-1 platform from Quanterix (Billerica, MA, USA) following the manufacturer's protocol using a cytokine 3-plex A advantage assay (101160). This assay was run using an on board 4-fold dilution and all samples were run in duplicate. Subjects were randomized into three runs, each of which included an 8-point standard curve, two manufacturer provided controls and three internally provided serum controls. Serum samples above the upper limit of detection were reanalyzed using an off-board 20-fold dilution.

Markers of endothelial injury were measured as follows. E-selectin, P-selectin, sICAM-3, and thrombomodulin were analyzed using the MSD Human Vascular Injury 1 Kit (K15135C, Meso Scale Diagnostics®, MD, USA). Syndecan-1 (LSEHSDC1) and von Willebrand Factor (LSEHVWF) were quantified using Invitrogen™ Human Elisa Kits (Invitrogen, CA, USA). The following protocol adaptations were made: Syndecan-1; all samples were diluted 20-fold, vWF; all samples were diluted 4000-fold. All samples were run on the same day split over 3 individual plates, each with an 8pt standard curve, two serum samples from healthy controls to serve as inter-plate controls, and a balance of control and HIBI patients all run in duplicate. Samples from an individual patient were all run on the same plate, using a randomized order for a total of 40 specimens per plate.

SUPPLEMENTAL TABLES

Online Table I. Patient characteristics and clinical data.

		**Normoxic (n=8)**	**Hypoxic (n=10)**
**Age (years)**	Median [IQR]	33 [22-54]	47 [42-52]
**Height (cm)**	Median [IQR]	172 [164-	184 [165-188]
**Weight (kg)**	Median [IQR]	68 [56-80]	85 [68-91]
**OHCA**	n (%)	6 (75)	9 (90)
**Witnessed**	n (%)	5 (62.5)	6 (60)
**Bystander CPR**	n (%)	5 (62.5)	8 (80)
**Initial Rhythm**	n (%)		
	PEA	7(87.5)	8(80)
	Asystole	0(0)	2(20)
	VF	1 (12.5)	0(0)
**Arrest etiology**	n (%)		
	Hypoxia	2(25)	4(40)
	Trauma	2(25)	0(0)
	Anaphylaxis	1(12.5)	0(0)
	VF	1(12.5)	0(0)
	Cocaine	1(12.5)	0(0)
	Hemorrhage	1(12.5)	0(0)
	Opioids	0(0)	2(20)
	Drowning	0(0)	2(20)
	Asthma	0(0)	1(10)
	Electrocution	0(0)	1(10)
**Time to ROSC (min)**	Median [IQR]	16 [13-24]	18 [17-26]
**Post ROSC GCS**	Median [IQR]	4 [4-6]	3 [3-3]
**Pupillary reflex**	n (%)	7 (87.5)	9 (90)
**Corneal reflex**	n (%)	7 (87.5)	7 (70)
**Motor Score**	Median [IQR]	1 [1-2]	1 [1-1.3]
**EEG waveform**	n (%)		
	Alpha rhythm	3 (37.5)	0 (0)
	Status epilepticus	2 (25)	1 (10)
	Severe encephalopathy	1 (12.5)	4 (40)
	GPEDs	1 (12.5)	1 (10)
	Myoclonus	0 (0)	1 (10)
	Modified burst suppression with Theta	1 (12.5)	2 (20)
	Theta	0 (0)	1 (10)
**SSEPs**	n (%)	0 (0)	0 (0)
**GW differentiation loss (CT)**	n (%)	1 (12.5)	7 (70)
**Restricted Diffusion (MRI)**	n (%)	2 (33)	4 (80)
**6 month CPC**	n (%)		

	1	4 (50)	1 (10)
	2	2 (25)	1 (10)
	3	0 (0)	0 (0)
	4	0 (0)	1 (1)
	5	2 (25)	7 (70)
**Delay from arrest to blood sample (hrs)**	Median [IQR]	19 [12-34]	28 [13-51]

CT, computed tomography; EEG, electroencephalography; GPED, Generalized periodic epileptiform discharge; MRI, magnetic resonance imaging; OHCA, out of hospital cardiac arrest; ROSC, return of spontaneous circulation; GCS, Glasgow coma scale; CPC, cerebral performance category; PEA, pulseless electrical activity; VF, ventricular fibrillation; IQR, interquartile range. There was no difference in the delay between cardiac arrest and the first blood sample for biomarker analyses (P=0.65). Note, one patient in the hypoxic group was lost to follow up. Not every patient received an MRI, therefore the % of patients noted with restricted diffusion is based upon the number of patients examined (6 for the normoxic patients and 5 for the hypoxic patients).

Online Table II. Arterial-to-Jugular venous gradients from biomarkers of neuronal, astroglial, and endothelial injury as well as neuro-inflammation.

		**Healthy Volunteers (N=14)**	**Normoxic HIBI patients (N=8)**	**Hypoxic HIBI patients (N=10)**
**GFAP**	(pg/mL)	-2.4 [-8.0 – 3.7]	-0.7 [-37.5 – 12.1]	-161 [-3695 – -75]
**NF-L**	(pg/mL)	-0.2 [-0.4 – 0.5]	0.4 [-3.1 – 23.0]	-231 [-370 – -11]
**Tau**	(pg/mL)	-0.05 [-0.16 – 0.01]	0.04 [-0.65 – 0.32]	-32 [-310 – -3]
**NSE**	(pg/mL)	-301 [-1454 – 271]	-182 [-3114 – 6331]	-14890 [-148813 – -3311]
**UCH-L1**	(pg/mL)	0.0 [-1.8 – 0.0]	-1.5 [-4.6 – 3.0]	-14.7 [-37.7 – -4.1]
**E-selectin**	(ng/mL)	0.15 [-0.28 – 0.70]	0.16 [-0.20 – 1.19]	0.12 [-1.6 – 5.3]
**P-selectin**	(ng/mL)	0.59 [-2.91 – 6.57]	0.13 [-7.9 – 4.50]	2.10 [-10.4 – 10.1]
**sICAM-3**	(ng/mL)	0.02 [-0.07 – 0.14]	-0.01 [-0.20 – 0.52]	-0.03 [-0.45 – 0.13]
**Thrombomodulin**	(ng/mL)	0.05 [-0.10 – 0.14]	-0.01 [-0.49 – 1.04]	0.13 [-0.20 – 0.80]
**vWF**	(ng/mL)	1380 [-1131 – 7875]	2643 [-3354 – 20918]	1365 [-13717 – 8596]
**Syndecan-1**	(pg/mL)	-149 [-239 – 1178]	-460 [-1360 – 245]	-268 [-834 – 117]
**IL-6**	(pg/mL)	-0.38 [-1.8 – -0.21]	2.6 [-3.6 – 19.7]	-10.3 [-43.0 – -4.2]
**IL-10**	(pg/mL)	-0.02 [-0.05 – 0.0]	0.35 [-1.6 – 2.3]	0.14 [-2.2 – 0.38]
**TNF-α**	(pg/mL)	-0.43 [-1.8 – -0.21]	0.09 [-0.35 – 0.15]	0.03 [-0.42 – 0.25]

Online Table III. Relationship between time to return of spontaneous circulation and the arterial-to-venous gradient for markers of neurovascular unit injury.

	Spearman's Rho	95% CI's	P-value
GFAP	-0.13	-0.60 to 0.40	0.61
NF-L	-0.11	-0.56 to 0.39	0.67
Tau	-0.38	-0.73 to 0.12	0.12
NSE	-0.19	-0.61 to 0.32	0.46
UCH-L1	-0.01	-0.49 to 0.47	0.97

Data represent the correlation between the time to return of spontaneous correlation and the cerebral release of each biomarker (i.e. A-v gradient). Significance testing was conducted with the non-parametric Spearman's correlation.

Online Table IV. Receiver operator curve characteristics for clinical labs and brain biomarkers to differentiate between brain hypoxia and normoxia in post cardiac arrest HIBI patients.

Clinical Laboratory Data (Arterial Blood Samples)						
	Admission		Peak		Median	
	AUC	P-value	AUC	P-value	AUC	P-value
Lactate	0.71±0.13	0.15	0.71±0.13	0.13	0.58±0.14	0.56
pH	0.68±0.13	0.20	0.64±0.14	0.31	0.73±0.13	0.11
HCO_3^-	0.57±0.15	0.63	0.56±0.14	0.66	0.53±0.14	0.82
Troponin	0.76±0.12	0.062	0.58±0.15	0.59	0.51±0.15	0.96
LDH	0.63±0.14	0.37	0.50±0.15	1.0	0.64±0.14	0.33
Creatinine	0.61±0.14	0.45	0.57±0.14	0.63	0.58±0.14	0.59
AST	0.53±0.15	0.86	0.54±0.14	0.79	0.51±0.14	0.93
ALT	0.51±0.15	0.93	0.60±0.14	0.48	0.50±0.14	1.0
Brain Biomarkers (Arterial Blood Samples)						
	Admission		Peak		Median	
	AUC	P-value	AUC	P-value	AUC	P-value
GFAP	0.85±0.10	**0.013**	0.89±0.08	**0.0059**	0.93±0.06	**0.0025**
NF-L	0.88±0.09	**0.0077**	0.93±0.06	**0.0025**	0.93±0.06	**0.0025**
Tau	0.90±0.07	**0.0045**	0.95±0.05	**0.0014**	0.95±0.05	**0.0014**
UCH-L1	0.81±0.11	**0.026**	0.85±0.10	**0.013**	0.86±0.10	**0.010**
NSE	0.80±0.11	**0.033**	0.79±0.11	**0.041**	0.76±0.12	0.062
Brain Biomarkers (A-v gradients)						
	Admission		Minimum		Median	
	AUC	P-value	AUC	P-value	AUC	P-value
GFAP	0.90±0.08	**0.0053**	0.89±0.09	**0.0071**	0.86±0.10	**0.012**
NF-L	0.76±0.12	0.062	0.79±0.12	**0.041**	0.86±0.10	**0.010**
Tau	0.90±0.09	**0.0045**	0.95±0.05	**0.0014**	0.83±0.11	**0.021**
UCH-L1	0.84±0.10	**0.016**	0.78±0.12	0.054	0.79±0.12	**0.043**
NSE	0.86±0.09	**0.010**	0.89±0.08	**0.0059**	0.85±0.09	**0.013**

Receiver operator characteristic analysis for admission, peak, and median data over the first 3 days of monitoring. N= 18 for all ROC curve data, except for fibrinogen where N=12 (4 normoxic and 8 hypoxic patients). ROC data are the area under the curve (AUC) ± standard error.

SUPPLEMENTAL FIGURES

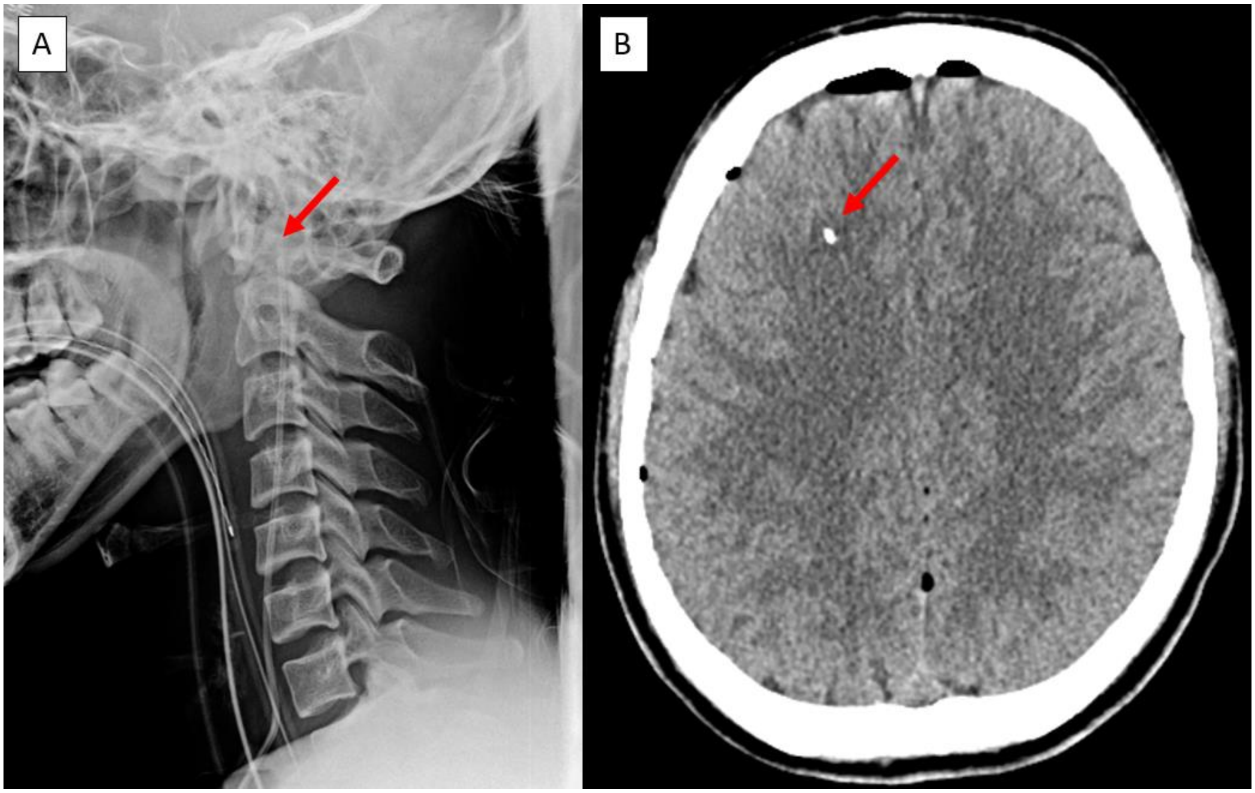

Online Figure I. Confirmation of the jugular bulb catheter and cranial access bolt. The position of the jugular bulb catheter tip (Panel A) and the cranial access bolt (Panel B) are denoted by red arrows.

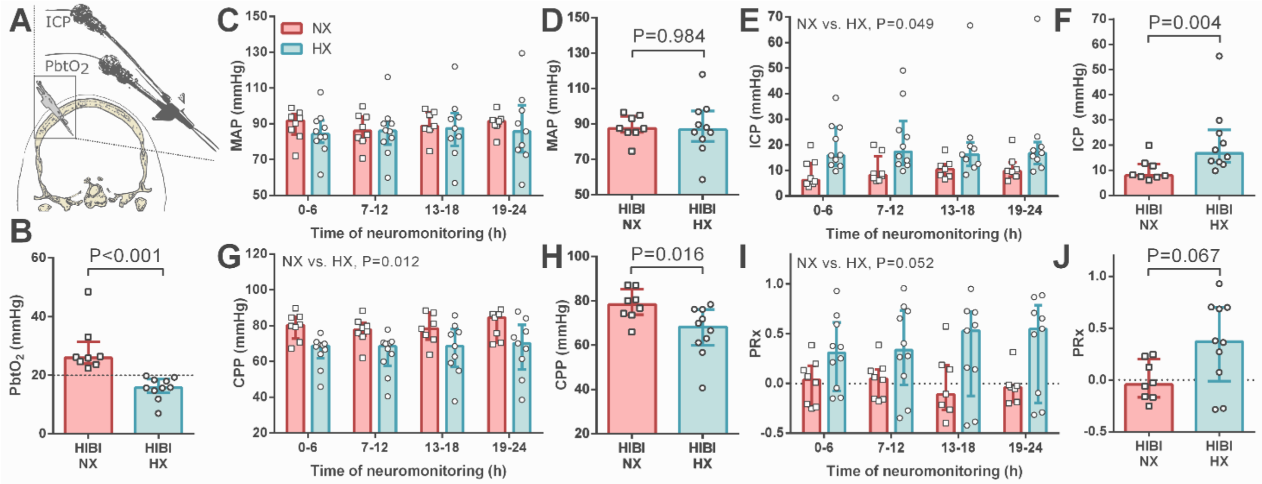

Online Figure II. Cerebrovascular physiology and brain tissue partial pressure of oxygen in hypoxic ischemic brain injury patients following cardiac arrest. Hypoxic ischemic brain injury (HIBI) patients exhibiting brain normoxia (NX), are presented as a red bars (median±IQR), while those exhibiting brain hypoxia (HX) are denoted by the cyan coloured bars. Individual patient data are overlaid for both groups. Mean arterial pressure (MAP: Panel A&B), intracranial pressure (ICP: Panel C&D), cerebral perfusion pressure (CPP; Panel E&F), and the pressure reactivity index (PRx; an index of cerebral autoregulation[15,55] Panel G&H) are depicted in 6 hour increments of neuromonitoring as well as their overall average values for the first 24hours of neuromonitoring combined. Importantly, to differentiate between brain hypoxia and normoxia and stratify our patients, a brain partial pressure of oxygen ($PbtO_2$; panel I) cut off of 20 mmHg was used. The 6 hour binned neuromonitroing data were assessed using linear mixed effects models using a compound symmetry co-variance structure with both time (0-6 vs. 7-12 vs. 13-18 vs. 19-24 hours) and brain oxygenation (normoxia vs. hypoxia) as fixed factors and patients as a random factor. Twenty-four hour mean data were compared using a Mann-Whitney U Test.

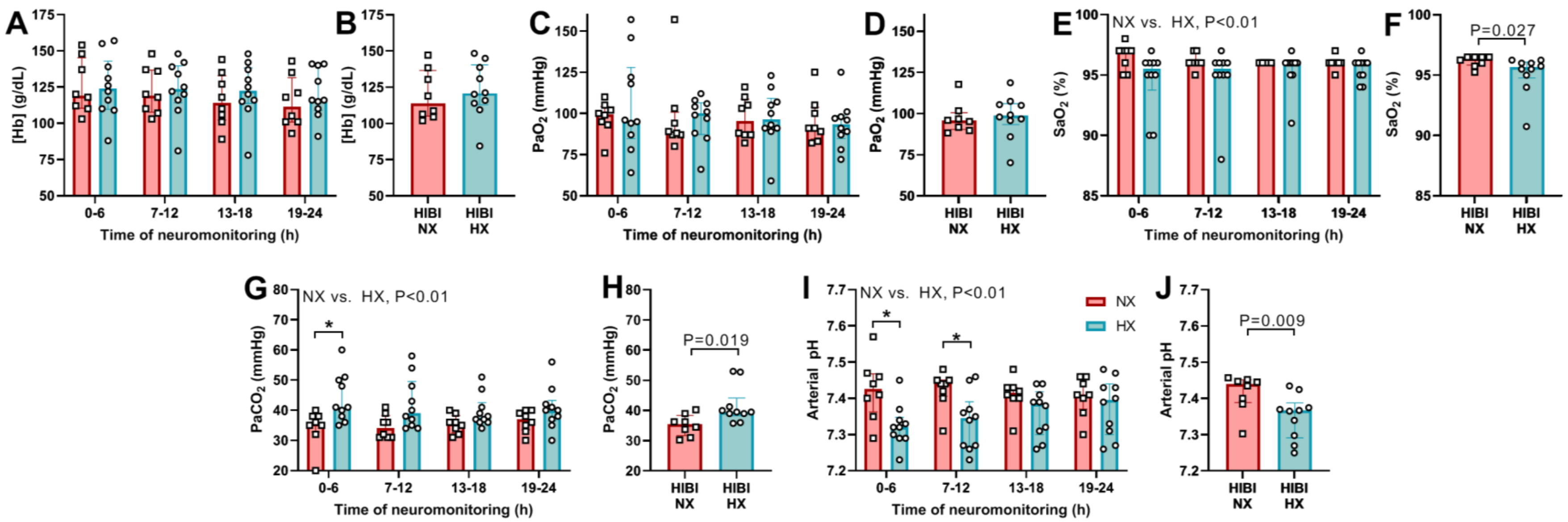

Online Figure III. Arterial blood gases in the hypoxic ischemic brain injury patients following cardiac arrest. Hypoxic ischemic brain injury (HIBI) patients exhibiting brain normoxia (NX), are presented as a red bars (median±IQR), while those exhibiting brain hypoxia (HX) are denoted by the cyan coloured bars. Individual patient data are overlaid for both groups. Hemoglobin concentration ([Hb]: Panel A&B), the partial pressure of arterial oxygen (PaO_2: Panel C&D), arterial oxygen saturation (SaO_2; Panel E&F), the partial pressure of arterial carbon dioxide ($PaCO_2$; Panel G&H), and arterial pH (Panel I&J) are depicted in 6 hour increments of neuromonitoring as well as their overall average values for the first 24hours of neuromonitoring combined. The 6 hour binned neuromonitroing data were assessed using linear mixed effects models using a compound symmetry co-variance structure with both time (0-6 vs. 7-12 vs. 13-18 vs. 19-24 hours) and brain oxygenation (normoxia vs. hypoxia) as fixed factors and patients as a random factor. Twenty-four hour mean data were compared using a Mann-Whitney U Test.

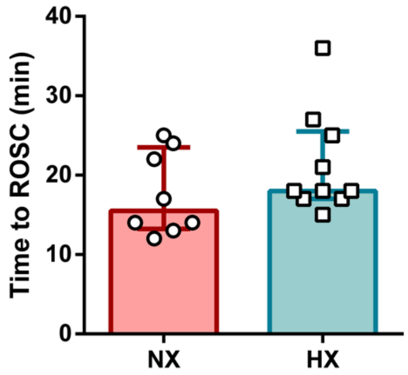

Online Figure IV. **Time to return of spontaneous circulation in post cardiac arrest patient with brain normoxia and hypoxia.** Hypoxic ischemic brain injury (HIBI) patients exhibiting brain normoxia (NX), are presented as a red bars (median±IQR), while those exhibiting brain hypoxia (HX) are denoted by the cyan coloured bars. Individual patient data are overlaid for both groups. Significance testing was conducted with a Mann Whitney U test. Time to ROSC was not different between groups (P=0.19).

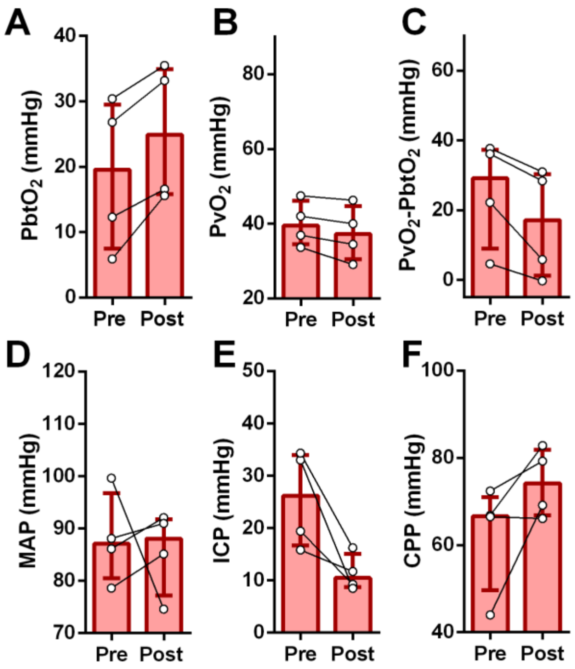

Online Figure V. Hypertonic saline infusion in cardiac arrest patients with brain normoxia. Hypoxic ischemic brain injury (HIBI) patients exhibiting brain normoxia (NX, n=4), are presented as a red bars (median±IQR). Individual patient data are overlaid. The one hour mean immediately pre to hypertonic saline infusion (pre) and the six hour mean immediately following infusion (post) are depicted for brain tissue partial pressure of oxygen ($PbtO_2$; Panel A), partial pressure of jugular bulb venous oxygen (PvO_2; Panel B), the PvO_2-$PbtO_2$ gradient (Panel C), mean arterial pressure (MAP; Panel D), intracranial pressure (ICP; Panel E), and cerebral perfusion pressure (CPP; Panel F). Only 4 normoxic HIBI patients received hypertonic saline based on clinical indication, thus while the data are presented for transparency no statistical analyses were completed.
