# Supplementary material for: Brain Hypoxia Is Associated With Neuroglial Injury in Humans Post–Cardiac Arrest
Source: Circ Res. 2021 Jul 21;129(5):583–97. doi: 10.1161/CIRCRESAHA.121.319157 (PMC8376277; doi:10.1161/CIRCRESAHA.121.319157)
Supplement: Supplementary file 2 [file res-129-583-s002.pdf]

Major Resources Table

In order to allow validation and replication of experiments, all essential research materials listed in the Methods should be included in the Major Resources Table below. Authors are encouraged to use public repositories for protocols, data, code, and other materials and provide persistent identifiers and/or links to repositories when available. Authors may add or delete rows as needed.

Animals (in vivo studies)

Species	Vendor or Source	Background Strain	Sex	Persistent ID / URL
N/A				

Genetically Modified Animals

	Species	Vendor or Source	Background Strain	Other Information	Persistent ID / URL
**Parent - Male**	N/A				
**Parent - Female**					

Antibodies

Target antigen	Vendor or Source	Catalog #	Working concentration	Lot # (preferred but not required)	Persistent ID / URL
N/A					

DNA/cDNA Clones

Clone Name	Sequence	Source / Repository	Persistent ID / URL
N/A			

Cultured Cells

Name	Vendor or Source	Sex (F, M, or unknown)	Persistent ID / URL
N/A			

Data & Code Availability

Description	Source / Repository	Persistent ID / URL
N/A		

Other

Biomarker Analysis Kits	Vendor or Source	Catalog #	URL
Glial Fibrillary Acidic Protein	Quanterix	102336	https://www.pblassaysci.com/sites/default/files/qtx_gfap_dis_102336.pdf
Neurofilament Light	Quanterix	103186	https://www.pblassaysci.com/sites/default/files/qtx_nf-l_adv_103186.pdf

DOI [to be added]

Total Tau	Quanterix	101552	https://www.chimera-biotec.com/fileadmin/public/images/Datasheets/Tau_item_101552_Chimera.pdf
Neuron Specific Enolase	Quanterix	102475	https://www.chimera-biotec.com/fileadmin/public/images/Datasheets/NSE_item_102475_Chimera.pdf
Ubiquitin carboxyl-terminal hydrolase L1	Quanterix	102343	https://www.chimera-biotec.com/fileadmin/public/images/Datasheets/UCH-L1_item_102343_Chimera.pdf
Interleukin-6; Interleukin 10; Tumor Necrosis Factor Alpha	Quanterix	101160	https://www.quanterix.com/wp-content/uploads/2020/12/Simoa_C3PA_Data_Sheet-SR-X.pdf
E-selectin; P-selectin; soluble intracellular adhesion molecule – 3; Thrombomodulin	Meso Scale Diagnostics	K15135C	https://www.mesoscale.com/en/products/human-vascular-injury-i-kit-k15135c/
Von Willebran Factor	Invitrogen	LSEHVWF	https://www.fishersci.ca/shop/products/human-vwf-elisa-kit-thermo-scientific-pierce/ehvwf
Syndecan-1	Invitrogen	LSEHSDC1	https://www.fishersci.ca/shop/products/human-syndecan-1-sdc1-elisa-kit-thermo-scientific-pierce/ehsdc1?searchHijack=true&searchTerm=EHSDC1&searchType=RAPID&matchedCatNo=EHSDC1

DOI [to be added]
