# Supplementary figures and images for: Brain Hypoxia Is Associated With Neuroglial Injury in Humans Post–Cardiac Arrest
Source: Circ Res. 2021 Jul 21;129(5):583–97. doi: 10.1161/CIRCRESAHA.121.319157 (PMC8376277; doi:10.1161/CIRCRESAHA.121.319157)

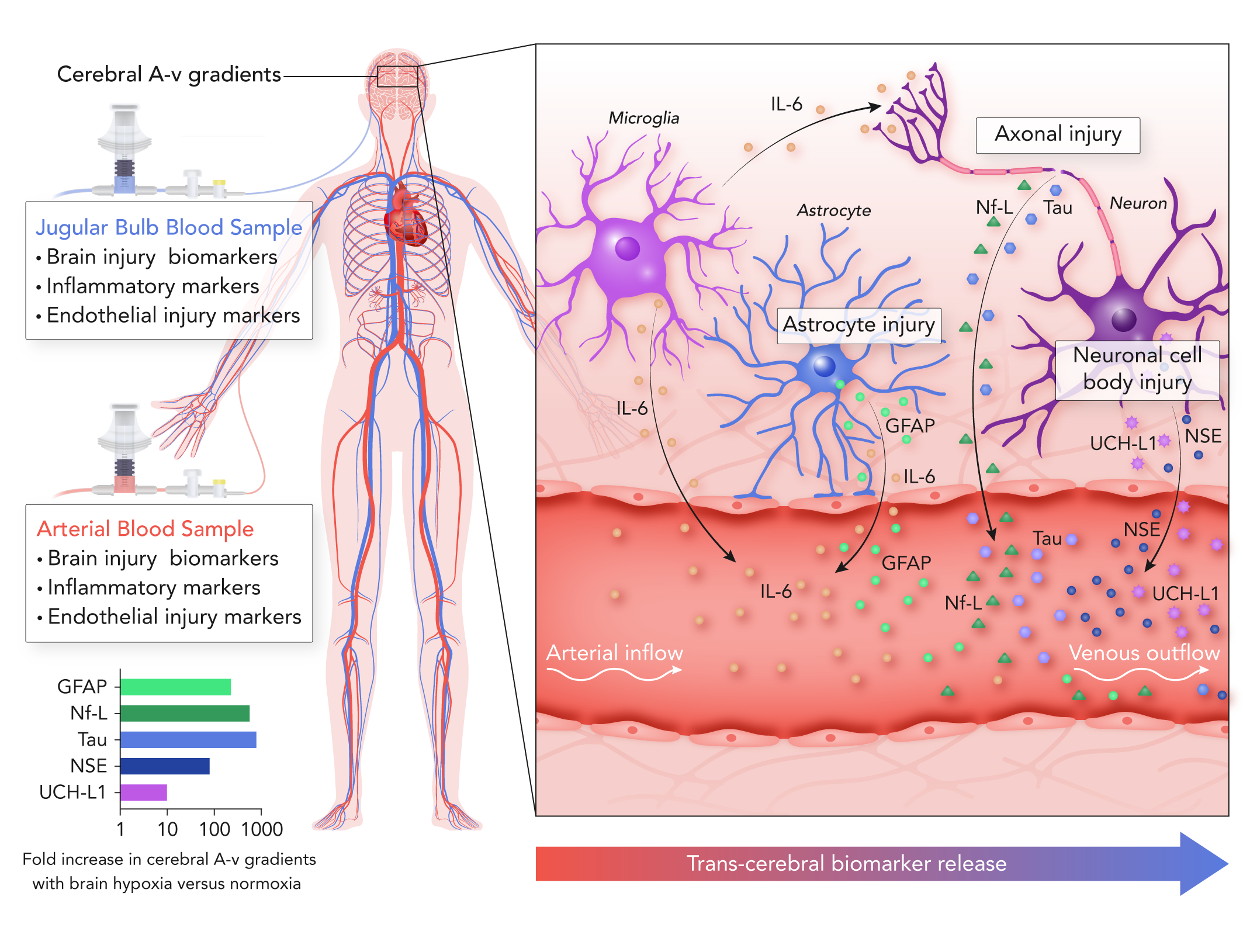

Supplement: Supplementary file 3 [file res-129-583-s003.tif]
